# Supplementary material for: Impact of clinical supervision on healthcare organisational outcomes: A mixed methods systematic review
Source: PLoS One. 2021 Nov 19;16(11):e0260156. doi: 10.1371/journal.pone.0260156 (PMC8604366; doi:10.1371/journal.pone.0260156)
Supplement: S1 Table — (DOCX) [file pone.0260156.s002.docx]

**Supplementary Table 1**. JBI Critical Appraisal Checklist for Randomised Controlled Trials

| **Study** | **1** | **2** | **3** | **4** | **5** | **6** | **7** | **8** | **9** | **10** | **11** | **12** | **13** |
| --- | --- | --- | --- | --- | --- | --- | --- | --- | --- | --- | --- | --- | --- |
| Wallbank  2010 | Unclear | Unclear | Unclear | No | No | Unclear | Unclear | Yes | Yes | Yes | Yes | No | Yes |

1 – Was true randomisation used for assignment of participants to treatment groups?

2 – Was allocation to groups concealed?

3 – Were treatment groups similar at the baseline?

4 – Were participants blinded to treatment assignment?

5 – Were those delivering treatment blind to treatment assignment?

6 – Were outcomes assessors blind to treatment assignment?

7 – Were treatment groups treated identically other than the intervention of interest?

8 – Was follow up complete and if not were differences between groups in terms of their follow up adequately described and analysed?

9 – Were participants analysed in the groups to which they were randomised?

10 – Were outcomes measured in the same way for the treatment groups?

11 – Were outcomes measured in a reliable way?

12 – Was appropriate statistical analysis used?

13 – Was the trial design appropriate for the topic, and any deviations from the standard RCT design accounted for in the conduct of the analysis?
