# Supplementary material for: Impact of clinical supervision on healthcare organisational outcomes: A mixed methods systematic review
Source: PLoS One. 2021 Nov 19;16(11):e0260156. doi: 10.1371/journal.pone.0260156 (PMC8604366; doi:10.1371/journal.pone.0260156)
Supplement: S2 Table — (DOCX) [file pone.0260156.s003.docx]

**Supplementary Table 2**. JBI Critical Appraisal Checklist for Quasi-Experimental Studies

| **Study** | **1** | **2** | **3** | **4** | **5** | **6** | **7** | **8** | **9** |
| --- | --- | --- | --- | --- | --- | --- | --- | --- | --- |
| Begat  1997 | Yes | Yes | Unclear | No | Yes | No | Yes | No | Yes |
| Begat  2005 | Yes | Unclear | Unclear | Yes | No | N/A | Yes | No | Yes |
| Berg  1994 | Yes | Yes | Unclear | Yes | Yes | No | Yes | Yes | Yes |
| Berg  1999 | Yes | Yes | Unclear | No | Yes | No | Yes | Yes | Yes |
| Cooper-Nurse  2018 | Yes | Unclear | Unclear | Yes | No | N/A | Yes | Yes | Yes |
| Eklund  2000 | Yes | Unclear | Unclear | Yes | No | N/A | Yes | Yes | Yes |
| Fischer  2013 | Yes | Unclear | Unclear | Yes | No | N/A | Yes | Yes | Unclear |
| Hallberg  1994 | Yes | Yes | Yes | No | Yes | Unclear | Yes | Yes | Yes |
| Koivu  2012 | Yes | Yes | Unclear | Yes | No | N/A | Yes | Yes | Yes |
| Livini  2012 | Yes | Yes | Yes | No | Yes | Yes | Yes | No | Yes |
| Love *  2017 | Yes | Unclear | Unclear | Yes | No | N/A | Yes | Yes | Yes |
| McAuliffe  2013 | Yes | Unclear | Unclear | Yes | No | N/A | Yes | Yes | Yes |
| Severinsson  1999 | Yes | Unclear | Unclear | Yes | No | N/A | Yes | No | Yes |
| Teasedale  2001 | Yes | Yes | Unclear | Yes | No | N/A | Yes | Yes | Yes |

* Quantitative component of the mixed methods study reviewed

1 - Is it clear in the study what is the ‘cause’ and what is the ‘effect’?

2 - Were the participants included in any comparisons similar?

3 - Were the participants included in any comparisons receiving similar treatment/care, other than the exposure or intervention of interest?

4 - Was there a control group?

5 - Were there multiple measurements of the outcome both pre and post the intervention/exposure?

6 - Was follow up complete and if not, were differences between groups in terms of their follow up adequately described and analyzed?

7 - Were the outcomes of participants included in any comparisons measured in the same way?

8 - Were outcomes measured in a reliable way?

9 - Was appropriate statistical analysis used?
