# Supplementary material for: Impact of clinical supervision on healthcare organisational outcomes: A mixed methods systematic review
Source: PLoS One. 2021 Nov 19;16(11):e0260156. doi: 10.1371/journal.pone.0260156 (PMC8604366; doi:10.1371/journal.pone.0260156)
Supplement: S3 Table — (DOCX) [file pone.0260156.s004.docx]

**Supplementary Table 3**. JBI Critical Appraisal Checklist for Analytical Cross Sectional Studies

| **Study** | **1** | **2** | **3** | **4** | **5** | **6** | **7** | **8** |
| --- | --- | --- | --- | --- | --- | --- | --- | --- |
| Ben-Porat 2011 | Yes | Yes | Yes | N/A | No | No | Yes | Yes |
| Berry  2019 | Yes | Yes | Yes | N/A | Yes | Yes | Yes | Yes |
| Best  2014 | Yes | Yes | Yes | N/A | Yes | Yes | Yes | Yes |
| Edwards  2006 | Yes | No | Yes | N/A | Yes | No | Yes | Yes |
| Hussein  2019 | Yes | Yes | Yes | N/A | Yes | Yes | Yes | Yes |
| Hyrkäs  2005 | Yes | Yes | Yes | N/A | Yes | No | Yes | No |
| Gonge  2011 | Yes | Yes | Yes | N/A | Yes | Yes | Yes | Yes |
| Kavanagh  2003 | Yes | Yes | Yes | N/A | Yes | Yes | Yes | Yes |
| Long  2014 | Yes | Yes | Yes | N/A | No | N/A | No | N/A |
| Nathanson  1992 | Yes | Yes | No | N/A | Yes | No | No | Yes |
| Saxby  2016 | Yes | Yes | Yes | N/A | Yes | No | Yes | Yes |
| Schroffel  1999 | Yes | No | No | N/A | Yes | No | Yes | Unclear |
| Severinsson  1996 | Yes | Yes | No | N/A | Yes | N/A | No | N/A |
| Webster  1999 | Yes | Yes | No | N/A | No | No | Yes | Yes |

* Quantitative component of the mixed methods study reviewed

1 - Were the criteria for inclusion in the sample clearly defined?

2 - Were the study subjects and the setting described in detail?

3 - Was the exposure (CS) measured in a valid and reliable way?

4 - Were objective, standard criteria used for measurement of the condition?

5 - Were confounding factors identified?

6 - Were strategies to deal with confounding factors stated?

7 - Were the outcomes measured in a valid and reliable way (Organisational Outcome)?

8 - Was appropriate statistical analysis used?
