# Supplementary material for: Impact of clinical supervision on healthcare organisational outcomes: A mixed methods systematic review
Source: PLoS One. 2021 Nov 19;16(11):e0260156. doi: 10.1371/journal.pone.0260156 (PMC8604366; doi:10.1371/journal.pone.0260156)
Supplement: S4 Table — (DOCX) [file pone.0260156.s005.docx]

**Supplementary Table 4**. JBI Critical Appraisal Checklist for Qualitative Studies (Including Qualitative Component of Mixed Methods Studies)

| **Question** | **Love et al 2017**  **(Mixed methods)** | **McCarron et al 2017**  **(Mixed methods)** | **White et al 1998** | **Ducat et al 2016** | **Saxby 2016**  **(Mixed methods)** |
| --- | --- | --- | --- | --- | --- |
| 1. Is there congruity between the stated philosophical perspective and the research methodology? | Y | N | Y | Y | Y |
| 2. Is there congruity between the research methodology and the research question or objectives? | Y | N | Y | Y | Y |
| 3. Is there congruity between the research methodology and the methods used to collect data? | Y | N | Y | Y | Y |
| 4. Is there congruity between the research methodology and the representation and analysis of data? | Y | N | Y | Y | Y |
| 5. Is there congruity between the research methodology and the interpretation of results? | Y | N | Y | Y | Y |
| 6. Is there a statement locating the researcher culturally or theoretically? | Y | Y | N | N | Y |
| 7. Is the influence of the researcher on the research, and vice- versa, addressed? | Y | Y | N | N | Y |
| 8. Are participants, and their voices, adequately represented? | Y | Y | Y | Y | Y |
| 9. Is the research ethical according to current criteria or, for recent studies, and is there evidence of ethical approval by an appropriate body? | Y | Y | Y | Y | Y |
| 10. Do the conclusions drawn in the research report flow from the analysis, or interpretation, of the data? | Y | Y | Y | Y | Y |

**Y: yes; N: No; U: Unclear**
