# Supplementary material for: Impact of clinical supervision on healthcare organisational outcomes: A mixed methods systematic review
Source: PLoS One. 2021 Nov 19;16(11):e0260156. doi: 10.1371/journal.pone.0260156 (PMC8604366; doi:10.1371/journal.pone.0260156)
Supplement: S5 Table — (DOCX) [file pone.0260156.s006.docx]

**Supplementary Table 5**. Results of studies investigating the effect of clinical supervision on organisational outcomes compared to control (no supervision)

| **Study** | **Design** | **n** | **Outcome** | **Result**  +VE SMD favours clinical supervision group  (Bold indicates statistical significance) |
| --- | --- | --- | --- | --- |
| **Burnout** | | | | |
| Berg  1994 | Pre/post | Supervision n=16  Control n=15 | **Maslach Burnout Inventory** |  |
|  |  |  | Emotional exhaustion frequency | SMD 0.49 (95%CI -0.22 to 1.21) |
|  |  |  | Emotional exhaustion intensity | SMD 0.63 (95%CI -0.09 to 1.36) |
|  |  |  | Depersonalisation frequency | SMD -0.15 (95%CI -0.86 to 0.55) |
|  |  |  | Depersonalisation intensity | SMD 0.30 (95%CI -0.41 to 1.00) |
|  |  |  | Personal accomplishment frequency | SMD 0.31 (95%CI -0.40 to 1.02) |
|  |  |  | Personal accomplishment intensity | SMD 0.22 (95%CI -0.48 to 0.93) |
|  |  |  | Total frequency | SMD 0.17 (95%CI -0.54 to 0.87) |
|  |  |  | Total intensity | SMD 0.40 (95%CI -0.31 to 1.11) |
| Cooper-Nurse  2018 | Cross sectional | Supervision (face-face) n=26  Supervision (hybrid) n=18  Control n=16 | **Maslach Burnout Inventory** |  |
|  |  |  | Emotional exhaustion | N/S^a^ |
|  |  |  | Depersonalisation | N/S^a^ |
|  |  |  | Personal accomplishment | N/S^a^ |
| Koivu  2012 | Cross sectional | Supervision=74  Control=156 | **Maslach Burnout Inventory** |  |
|  |  |  | Overall burnout | SMD -0.13 (95%CI -0.43 to 0.18) |
|  |  |  | Exhaustion | SMD -0.23 (95%CI -0.54 to 0.07) |
|  |  |  | Cynicism | SMD -0.12 (95%CI -0.44 to 0.19) |
|  |  |  | Professional efficacy | **SMD 0.41 (95%CI 0.10 to 0.72)** |
| Fischer  2013 | Cross sectional | Supervision n=64  Control n=68 | **Maslach Burnout Inventory** | N/S^a^ |
| Teasedale  2001 | Cross sectional | Supervision n=96  Control n=115 | **Maslach Burnout Inventory** |  |
|  |  |  | Emotional exhaustion | SMD 0.17 (95%CI -0.11 to 0.45) |
|  |  |  | Depersonalisation | SMD 0.27 (95%CI -0.01 to 0.55) |
|  |  |  | Personal accomplishment | SMD 0.21 (95%CI -0.06 to 0.50) |
| Wallbank  2010 | RCT | Supervision n=15  Control n=15 | **Professional Quality of Life Scale** |  |
|  |  |  | Burnout scale | **SMD 2.07 (95%CI 1.18 to 2.96)** |
| **Job Retention** | | | | |
| McAuliffe  2013 | Cross sectional | Cohort 1  Supervision n=188  Control n=155 | **Intention to leave^b^** | **SMD 0.38 (95%CI 0.16 to 0.59)** |
|  |  | Cohort 2  Supervision n=287  Control n=112 | **Intention to leave^b^** | **SMD 0.32 (95%CI 0.11 to 0.53)** |
|  |  | Cohort 3  Supervision n=309  Control n=46 | **Intention to leave^b^** | **SMD 0.42 (95%CI 0.11 to 0.73)** |
| **Job Satisfaction** | | | | |
| Cooper-Nurse  2018 | Cross sectional | Supervision (face-face) n=26  Supervision (hybrid) n=18  Control n=16 | **Abridged Job Descriptive Index** | N/S^a^ |
|  |  |  |  |  |
|  |  |  |  |  |
| Eklund  2000 | Cross sectional | Discipline supervision n=108  Control n=183 | **Job satisfaction questionnaire** |  |
|  |  |  | General job satisfaction | p=0.38^a^ |
|  |  |  | Communication and co-operation | p=0.73^a^ |
|  |  |  | Managerial feedback | p=0.41^a^ |
|  |  |  | Patient’s influence on care | p=0.86^a^ |
|  |  |  | Relatives influence on care | p=0.99^a^ |
| Eklund  2000 | Cross sectional | Multi-professional  supervision n=169  Control n=122 | **Job satisfaction questionnaire** |  |
|  |  |  | General job satisfaction | p=0.05^a^ |
|  |  |  | Communication and co-operation | **p=0.001^a^** |
|  |  |  | Managerial feedback | **p=0.02^a^** |
|  |  |  | Patient’s influence on care | p=0.13^a^ |
|  |  |  | Relatives influence on care | p=0.74^a^ |
| Love  2017 | Cross sectional | Supervision n=71  Control n=47 | **Nursing Workplace Satisfaction Questionnaire** |  |
|  |  |  | Intrinsic job satisfaction | SMD 0.34 (95%CI -0.03 to 0.72) |
|  |  |  | Extrinsic job satisfaction | SMD 0.27 (95%CI -0.11 to 0.64) |
|  |  |  | Relational job satisfaction | SMD 0.24 (95%CI -0.13 to 0.61) |
|  |  |  | Total job satisfaction | SMD 0.35 (95%CI -0.02 to 0.72) |
| McAuliffe  2013 | Cross sectional | Cohort 1  Supervision n=188  Control n=155 | **Job satisfaction scale** | **SMD 0.31 (95%CI 0.09 to 0.52)** |
|  |  | Cohort 2  Supervision n=287  Control n=112 | **Job satisfaction scale** | **SMD 0.45 (95%CI 0.23 to 0.67)** |
|  |  | Cohort 3  Supervision n=309  Control n=46 | **Job satisfaction scale** | SMD 0.13 (95%CI -0.18 to 0.44) |
| **Well-being** | | | | |
| Begat  2005 | Cross sectional | Supervision n=22  Control n=49 | **Well-being Questionnaire^b^** |  |
|  |  |  | Physical symptoms and anxiety | **SMD 0.54 (95%CI 0.07 to 1.01)** |
|  |  |  | Feelings of not being in control | **SMD 0.52 (95%CI 0.04 to 0.99)** |
| Berg  1994 | Pre/post | Supervision n=16  Control n=15 | **The Tedium Measure**  Total score | SMD 0.50 (95%CI -0.22 to 1.21) |
| Koivu  2012 | Cross sectional | Supervision=74  Control=156 | **General Health Questionnaire-12** |  |
|  |  |  | Self-rated health | SMD -0.08 (95%CI -0.41 to 0.26) |
|  |  |  | Psychological distress | SMD -0.24 (95%CI -0.58 to 0.10) |
| Wallbank  2010 | RCT | Supervision n=15  Control n=15 | **Impact of Events Scale** | **SMD 3.15 (95%CI 2.08 to 4.22)** |
|  |  |  | **Professional Quality of Life Scale** |  |
|  |  |  | Compassion fatigue score | **SMD 0.84 (95%CI 0.09 to 1.58)** |
|  |  |  | Compassion satisfaction score | **SMD 1.03 (95%CI 0.27 to 1.79)** |
|  |  |  | Burnout score | **SMD 2.07 (95%CI 1.18 to 2.96)** |
| **Workplace Environment** | | | | |
| Begat  2005 | Cross sectional | Supervision n=22  Control n=49 | **Workplace environment questionnaire** | N/S^a^ |
| Berg  1994 | Pre/post | Supervision n=16  Control n=15 | **Creative Climate Questionnaire** |  |
|  |  |  | Challenge | SMD 0.48 (95%CI -0.23 to 1.20) |
|  |  |  | Freedom | SMD 0.50 (95%CI -0.18 to 1.17) |
|  |  |  | Idea-support | **SMD 0.96 (95%CI 0.22 to 1.71)** |
|  |  |  | Trust | **SMD 0.05 (95%CI 0.05 to 1.51)** |
|  |  |  | Dynamism | SMD 0.66 (95%CI -0.06 to 1.38) |
|  |  |  | Playfulness | **SMD 0.84 (95%CI 0.11 to 1.58)** |
|  |  |  | Debates | **SMD 0.84 (95%CI 0.11 to 1.58)** |
|  |  |  | Conflicts | **SMD 0.85 (95%CI 0.12 to 1.59)** |
|  |  |  | Risk-taking | SMD 0.56 (95%CI -0.16 to 1.28) |
|  |  |  | Idea-time | SMD 0.52 (95%CI -0.20 to 1.23) |
| Koivu  2012 | Cross sectional | Supervision=74  Control=156 | **The Nordic Questionnaire for Psychological and Social Factors** |  |
|  |  |  | Control at work | **SMD 0.36 (95%CI 0.08 to 0.64)** |
|  |  |  | Control of decisions | **SMD 0.31 (95%CI 0.03 to 0.58)** |
|  |  |  | Control of pacing | **SMD 0.33 (95%CI 0.05 to 0.61)** |
|  |  |  | Positive challenges | SMD 0.18 (95%CI -0.10 to 0.46) |
|  |  |  | Fair leadership | SMD 0.26 (95%CI -0.01 to 0.54) |
|  |  |  | Organisational culture and climate | SMD 0.18 (95%CI -0.10 to 0.46) |
|  |  |  | Social climate | SMD 0.18 (95%CI -0.10 to 0.46) |
|  |  |  | Equality | SMD 0.23 (95%CI -0.05 to 0.50) |
|  |  |  | Human resource primary | SMD 0.15 (95%CI -0.13 to 0.42) |
|  |  |  | Perception of group work | **SMD 0.37 (95%CI 0.09 to 0.65)** |
|  |  |  | Feedback on the quality of work | **SMD 0.50 (95%CI 0.22 to 0.78)** |
|  |  |  | Commitment to the organisation | SMD 0.25 (95%CI -0.02 to 0.53) |
|  |  |  | Intrinsic motivation | SMD 0.27 (95%CI -0.01 to 0.55) |
|  |  |  | Interaction between work and private life | **SMD -0.36 (95%CI -0.63 to -0.08)** |
| Severinsson  1999 | Cross sectional | Supervision n=94  Control n=64 | **Work environment measure^b^** |  |
|  |  |  | Total score | **SMD -0.5 (95%CI -0.82 to -0.18)** |
|  |  |  | Relation to superiors and colleagues | SMD -0.3 (95%CI -0.62 to 0.02) |
|  |  |  | Stress | SMD -0.26 (95%CI -0.58 to 0.06) |
|  |  |  | Engagement | **SMD -0.63 (95%CI -0.95 to -0.30)** |
|  |  |  | Perceived anxiety | SMD 0.13 (95%CI -0.18 to 0.45) |
|  |  |  | Physical and mental problems | SMD -0.21 (95%CI -0.53 to 0.11) |
| Teasdale  2001 | Cross sectional | Supervision n=96  Control n=115 | **Nursing in Context Questionnaire** |  |
|  |  |  | Listening and supportive management | **SMD 0.61 (95%CI 0.32 to 0.89)** |
|  |  |  | Coping at work | **SMD 0.39 (0.13 to 0.65)** |
|  |  |  | Accessing support | **SMD 0.48 (95%CI 0.20 to 0.76)** |

a – no measure of effect provided; b – outcome measure not validated; N/S – no significant difference between groups; RCT – randomised controlled trial.
