# Supplementary material for: Impact of clinical supervision on healthcare organisational outcomes: A mixed methods systematic review
Source: PLoS One. 2021 Nov 19;16(11):e0260156. doi: 10.1371/journal.pone.0260156 (PMC8604366; doi:10.1371/journal.pone.0260156)
Supplement: S6 Table — (DOCX) [file pone.0260156.s007.docx]

**Supplementary Table 6**. Results of studies investigating the effect of clinical supervision on organisational outcomes pre/post implementation

| **Study** | **Design** | **n** | **Outcome** | **Result**  +VE SMD favours post-supervision group  (Bold indicates statistical significance) |
| --- | --- | --- | --- | --- |
| **Burnout** | | | | |
| Berg  1994 | Pre/post | Baseline n=19  Follow-up n=16 | **Maslach Burnout Inventory** |  |
|  |  |  | Emotional exhaustion frequency | SMD 0.35 (95%CI -0.32 to 1.02) |
|  |  |  | Emotional exhaustion intensity | SMD 0.45 (95%CI -0.22 to 1.13) |
|  |  |  | Depersonalisation frequency | SMD 0.23 (95%CI -0.44 to 0.90) |
|  |  |  | Depersonalisation intensity | SMD 0.22 (95%CI -0.45 to 0.89) |
|  |  |  | Personal accomplishment frequency | **SMD 0.98 (95%CI 0.28 to 1.69)** |
|  |  |  | Personal accomplishment intensity | **SMD 1.11 (95%CI 0.40 to 1.82)** |
|  |  |  | Total frequency | SMD 0.67 (95%CI -0.03 to 1.36) |
|  |  |  | Total intensity | **SMD 0.77 (95%CI 0.08 to 1.46)** |
| Hallberg  1994 | Pre/post | Baseline n=11  Follow-up n=11 | **Maslach Burnout Inventory** |  |
|  |  |  | Emotional exhaustion frequency | SMD 0 (95%CI -0.84 to 0.84) |
|  |  |  | Emotional exhaustion intensity | SMD -0.21 (95%CI -1.05 to 0.63) |
|  |  |  | Depersonalisation frequency | SMD 0 (95%CI -0.84 to 0.84) |
|  |  |  | Depersonalisation intensity | SMD -0.65 (95%CI -1.51 to 0.20) |
|  |  |  | Personal accomplishment frequency | SMD 0.21 (95%CI -0.63 to 1.05) |
|  |  |  | Personal accomplishment intensity | SMD 0.32 (95%CI -0.53 to 1.16) |
| Livini  2012 | Pre/post | Baseline n=37  Follow-up=37 | **Maslach Burnout Inventory** | **SMD -0.67 (95%CI -1.13 to -0.20)** |
| Wallbank  2010 | RCT | Baseline n=15  Follow-up n=15 | **Professional Quality of Life Scale** |  |
|  |  |  | Burnout scale | **SMD 2.06 (95%CI 1.18 to 2.95)** |
| **Job satisfaction** | | | | |
| Berg 1994 | Pre/post | Baseline n=19  Follow-up n=16 | **Satisfaction with Nursing Care and Work** |  |
|  |  |  | Work means development and stimulation | **P<0.05^a,b^** |
|  |  |  | Satisfactory responsibility and quality of care | **P<0.05^a,b^** |
|  |  |  | Patients and family are well informed | P>0.05^a^ |
|  |  |  | Positive view of individualized care, supervision and workload | P>0.05^a^ |
|  |  |  | Satisfactory cooperation and comfort | **P<0.001^a,b^** |
|  |  |  | No time and energy for emotional involvement in patients | P>0.05^a^ |
|  |  |  | Too poor knowledge about families, their life stories, worries about future | **P<0.01^a,b^** |
|  |  |  | Important that families participate, that nurses can be sympathetic and that they share the work | P>0.05^a^ |
|  |  |  |  |  |
| Berg  1999 | Pre/post | Baseline n=22  Follow up n=18 | **Satisfaction with Nursing Care and Work Conditions** |  |
|  |  |  | Atmosphere/Development | SMD 0.33 (95%CI -0.30 to 0.96) |
|  |  |  | Satisfaction | SMD 0.43 (95%CI -0.20 to 1.06) |
|  |  |  | Flexibility | SMD 0.18 (95%CI -0.44 to 0.81) |
|  |  |  | Commitment | SMD 0.06 (95%CI -0.56 to 0.68) |
|  |  |  | Involvement | SMD 0.11 (95%CI -0.51 to 0.74) |
| Hallberg  1994 | Pre/post | Baseline n=11  Follow-up n=11 | **Satisfaction with Nursing Care and Work** |  |
|  |  |  | Work means development and stimulation | SMD 0.10 (95%CI -0.74 to 0.94) |
|  |  |  | Satisfactory responsibility and quality of care | SMD 0.75 (95%CI -0.11 to 1.62) |
|  |  |  | Patients and family are well informed | SMD 0.55 (95%CI -0.30 to 1.40) |
|  |  |  | Positive view of individualized care, supervision and workload | SMD -0.05 (95%CI -0.89 to 0.78) |
|  |  |  | Satisfactory cooperation and comfort | **SMD 1.14 (95%CI 0.24 to 2.04)** |
|  |  |  | No time and energy for emotional involvement in patients | SMD 0.20 (95%CI -0.64 to 1.03) |
|  |  |  | Too poor knowledge about families, their life stories, worries about future | SMD 0.25 (95%CI -0.59 to 1.08) |
|  |  |  | Important that families participate, that nurses can be sympathetic and that they share the work | SMD 0.06 (95%CI -0.77 to 0.90) |
| Livini  2012 | Pre/post | Baseline n=37  Follow-up=37 | **Intrinsic Job Satisfaction Scale** | P=0.84^a^ |
| **Well-being** | | | | |
| Berg  1994 | Pre/post | Baseline n=19  Follow-up n=16 | **Tedium Measure**  Total score | **SMD 0.72 (95%CI 0.03 to 1.40)** |
| Berg  1999 | Pre/post | Baseline n=22  Follow up n=18 | **Sense of Coherence Scale**  **Work-related Strain Inventory** | SMD 0.35 (95%CI -0.28 to 0.98)  SMD 0.36 (95%CI -0.27 to 0.98) |
| Hallberg  1994 | Pre/post | Baseline n=29  Follow-up n=32 | **Tedium Measure**  Total score  Physical exhaustion  Emotional exhaustion  Mental Exhaustion | SMD 0.44 (95%CI -0.40 to 1.29)  SMD 0.46 (95%CI -0.39 to 1.31)  SMD 0.31 (95%CI -0.53 to 1.15)  SMD 0.25 (95%CI -0.59 to 1.83) |
| Livini  2012 | Pre/post | Baseline n=37  Follow-up=37 | **Scales of Psychological Well-being** | **SMD -0.82 (95%CI -1.30 to -0.35)** |
| Wallbank  2010 | RCT | N/A | **Impact of Events Scale** | **SMD 2.66 (95%CI 1.68 to 3.64)** |
|  |  |  | **Professional Quality of Life Scale** |  |
|  |  |  | Compassion fatigue score | **SMD 0.76 (95%CI 0.02 to 1.50)** |
|  |  |  | Compassion satisfaction score | **SMD 0.91 (95%CI 0.15 to 1.66)** |
|  |  |  | Burnout score | **SMD 2.06 (95%CI 1.18 to 2.95)** |
| **Work environment** | | | | |
| Begat  1997 | Pre/post | Baseline n=29  Follow-up n=32 | **Nurses’ view of working milieu^c^** |  |
|  |  |  | Commitment | N/A |
|  |  |  | Workload | N/A |
|  |  |  | Lack of time | SMD -0.27 (95%CI -0.77 to 0.23) |
|  |  |  | Possibilities of others talking over one’s tasks | SMD -0.14 (95%CI -0.64 to 0.37) |
|  |  |  | Overtime worked | N/A |
|  |  |  | Degree of fatigue | SMD -0.38 (95%CI -0.88 to 0.13) |
|  |  |  | Variation of work tasks | N/A |
|  |  |  | Incompatible demands | SMD -0.29 (95%CI -0.80 to 0.21) |
|  |  |  | Degree of difficulties | SMD 0.30 (95%CI -0.21 to 0.80) |
|  |  |  | Very difficult tasks | SMD -0.15 (95%CI -0.65 to 0.65) |
|  |  |  | Support from supervisors | SMD 0.08 (95%CI -0.43 to 0.58) |
|  |  |  | Support from colleagues | SMD 0.03 (95%CI -0.48 to 0.53) |
|  |  |  | Lack of information from supervisors | SMD 0.38 (95%CI -0.88 to 0.13) |
|  |  |  | Doubts as to how to solve one’s tasks | SMD 0.33 (95%CI -0.18 to 0.83) |
|  |  |  | Responsibility |  |
|  |  |  | Others’ health/life | SMD 0.16 (95%CI -0.35 to 0.66) |
|  |  |  | Criticism from superiors | SMD -0.30 (95%CI -0.80 to 0.21) |
|  |  |  | Criticism from colleagues | **SMD -0.84 (95%CI -1.36 to 0.32)** |
|  |  |  | Communication possibilities | SMD -0.15 (95%CI -0.66 to 0.35) |
|  |  |  | Getting on well with colleagues | N/A |
|  |  |  | Getting on well with superiors | SMD -0.20 (95%CI -0.71 to 0.30) |
|  |  |  | Different opinions | SMD 0.28 (95%CI -0.22 to 0.79) |
|  |  |  | Colleagues’ estimation of one’s work | N/A |
|  |  |  | Superiors’ estimation of one’s work | N/A |
|  |  |  | Supervisors’ confirmation of one’s work | N/A |
|  |  |  | Confirmation/disconfirmation of one’s work | **SMD 0.91 (95%CI 0.38 to 1.4)** |
|  |  |  | Possibility of influencing one’s tasks | SMD 0.49 (95%CI -0.02 to 1.0) |
|  |  |  | Possibility of choosing quicker method | SMD 0.06 (95%CI -0.45 to 0.56) |
|  |  |  | Satisfaction with information | **SMD 0.59 (95%CI 0.07 to 1.10)** |
|  |  |  | Information about changes | SMD 0.26 (95%CI -0.24 to 0.77) |
| Berg  1994 | Pre/post | Baseline n=19  Follow-up n=16 | **Creative Climate Questionnaire** |  |
|  |  |  | Challenge | SMD 0.45 (95%CI -0.22 to 1.12) |
|  |  |  | Freedom | **SMD 0.99 (95%CI 0.29 to 1.70)** |
|  |  |  | Idea-support | **SMD 1.20 (95%CI 0.48 to 1.92)** |
|  |  |  | Trust | **SMD 0.80 (95%CI 0.11 to 1.49)** |
|  |  |  | Dynamism | **SMD 1.16 (95%CI 0.40 to 1.83)** |
|  |  |  | Playfulness | **SMD 0.86 (95%CI 0.16 to 1.55)** |
|  |  |  | Debates | **SMD 0.75 (95%CI 0.06 to 1.44)** |
|  |  |  | Conflicts | SMD 0.54 (95%CI -0.14 to 1.22) |
|  |  |  | Risk-taking | **SMD 1.14 (95%CI 0.43 to 1.86)** |
|  |  |  | Idea-time | **SMD 0.89 (95%CI 0.20 to 1.59)** |
| Berg  1999 | Pre/post | Baseline n=22  Follow up n=18 | **Creative Climate Questionnaire** |  |
|  |  |  | Challenge | SMD -0.19 (95%CI -0.82 to 0.43) |
|  |  |  | Freedom | SMD 0.2 (95%CI -0.42 to 0.82) |
|  |  |  | Idea-support | SMD 0.19 (95%CI -0.43 to 0.82) |
|  |  |  | Trust | SMD 0.54 (95%CI -0.10 to 1.17) |
|  |  |  | Dynamism | SMD 0.36 (95%CI -0.27 to 0.99) |
|  |  |  | Playfulness | SMD 0.54 (95%CI -0.01 to 1.17) |
|  |  |  | Debates | SMD 0.15 (95%CI -0.47 to 0.78) |
|  |  |  | Conflicts | SMD 0.54 (95%CI -0.10 to 1.17) |
|  |  |  | Risk-taking | SMD 0.2 (95%CI -0.42 to 0.82) |
|  |  |  | Idea-time | **SMD 0.72 (95%CI 0.08 to 1.36)** |

a – no measure of effect provided; b – result favoured post-supervision group; c – outcome measure not validated; N/A – not available due to unclear direction of effect (P>0.05); RCT – randomised controlled trial. NB: Maslach Burnout Inventory scores not available for each dimension in Livini et al. 2012.
