# Supplementary material for: Impact of clinical supervision on healthcare organisational outcomes: A mixed methods systematic review
Source: PLoS One. 2021 Nov 19;16(11):e0260156. doi: 10.1371/journal.pone.0260156 (PMC8604366; doi:10.1371/journal.pone.0260156)
Supplement: S7 Table — (DOCX) [file pone.0260156.s008.docx]

**Supplementary Table 7**. Results of studies investigating the association between effectiveness of clinical supervision and organisational outcomes

| **Study** | **Design** | **n** | **Measure of effectiveness** | **Outcome** | **Result**  (Bold indicates statistical significance) |
| --- | --- | --- | --- | --- | --- |
| **Burnout** | | | | | |
| Berry  2019 | Cross sectional | 137 | MCSS-26 | **Maslach Burnout Inventory** |  |
|  |  |  |  | Emotional Exhaustion | r= -0.14 |
|  |  |  |  | Depersonalisation | **r= -0.22** |
|  |  |  |  | Personal Accomplishment | r=-0.14 |
| Edwards  2006 | Cross sectional | 208 | MCSS | **Maslach Burnout Inventory** |  |
|  |  |  |  | Emotional Exhaustion | **r= -0.15** |
|  |  |  |  | Depersonalisation | **r= -0.22** |
|  |  |  |  | Personal Accomplishment | No correlation^a^ |
| Gonge  2011 | Cross sectional | 136 | MCSS | **Maslach Burnout Inventory** |  |
|  |  |  |  | Emotional Exhaustion | **β= -0.03 (95%CI -0.05 to -0.01)** |
|  |  |  |  | Depersonalisation | **β= -0.03 (95%CI -0.05 to -0.01)** |
|  |  |  |  | Personal Accomplishment | β= -0.01 (95%CI -0.03 to 0.01) |
| Hyrkäs  2005 | Cross sectional | 500 | MCSS | **Maslach Burnout Inventory** |  |
|  |  |  |  | Emotional Exhaustion^c^ | OR 1.30 (95%CI 0.91 to 1.85) |
|  |  |  |  | Depersonalisation^c^ | **OR 0.61 (95%CI 0.42 to 0.87)** |
|  |  |  |  | Personal Accomplishment^c^ | **OR 3.95 (95%CI 2.68 to 5.82)** |
| Saxby  2016 | Cross sectional | 63 | MCSS-26 | **Maslach Burnout Inventory** |  |
|  |  |  |  | Emotional Exhaustion^d^ | **SMD -0.62 (95%CI -1.20 to -0.05)** |
|  |  |  |  | Depersonalisation^d^ | SMD -0.48 (95%CI -1.06 to 0.09) |
|  |  |  |  | Personal Accomplishment^e^ | **SMD -0.68 (95%CI -1.19 to -0.17)** |
| **Job Retention** | | | | | |
| Hussein  2019 | Cross sectional | 87  (All wards) | MCSS-26 | **Modified Nurse Retention Index^f^** | OR 1.69 (95%CI 0.55 to 5.26) |
| Hussein  2019 | Cross sectional | 36  (Critical care) | MCSS-26 | **Modified Nurse Retention Index^f^** | **OR 3.84 (95%CI 1.32 to 11.11)** |
| Saxby  2016 | Cross sectional | 65 | MCSS-26 | **Intention to leave scale^e^** | **SMD 0.54 (95%CI 0.04 to 1.04)** |
| **Job Satisfaction** | | | | | |
| Best  2014 | Cross sectional | 43 | MCSS-26 restorative sub-scale | **Client Evaluation of Self and Treatment** | **β=0.19 (95%CI 0.06 to 0.31)** |
|  |  |  | MCSS-26 formative sub-scale | **Client Evaluation of Self and Treatment** | No association^a^ |
|  |  |  | MCSS-26 normative sub-scale | **Client Evaluation of Self and Treatment** | No association^a^ |
| Kavanagh  2003 | Cross sectional | 199 | Receipt of effective clinical supervision  (Likert Scale)^b^ | **Hoppock Job Satisfaction Measure** | **r= -0.17** |
|  |  |  |  |  | β= -0.15 (95%CI -0.71 to 0.41) |
| Gonge  2011 | Cross sectional | 136 | MCSS | **Copenhagen Psychosocial Questionnaire** |  |
|  |  |  |  | Job satisfaction scale | **β=0.02 (95%CI 0 to 0.03)** |
| Hyrkäs  2005 | Cross sectional | 501 | MCSS | **Minnesota Job Satisfaction Scale** |  |
|  |  |  |  | Extrinsic job satisfaction^c^ | OR 1.00 (95%CI 0.70 to 1.43) |
|  |  |  |  | Intrinsic job satisfaction^c^ | **OR 2.78 (95%CI 1.92 to 4)** |
|  |  |  |  | Total job satisfaction^c^ | **OR 1.69 (95%CI 1.18 to 2.44)** |
| **Well-being** | | | | | |
| Gonge  2011 | Cross sectional | 136 | MCSS | **Copenhagen Psychosocial Questionnaire** |  |
|  |  |  |  | Stress scale | **β=0.02 (95%CI 0 to 0.04)** |
|  |  |  |  | **36-Item Short Form Survey (SF-36)** |  |
|  |  |  |  | General Health | β=0.01 (95%CI 0.02 to 0.04) |
|  |  |  |  | Vitality | **β=0.04 (95%CI 0.01 to 0.06)** |
|  |  |  |  | Mental Health | β=0.01 (95%CI -0.01 to 0.04) |
| **Work Environment** | | | | | |
| Best  2014 | Cross sectional | 43 | MCSS-26 restorative sub-scale | **Organizational Readiness for Change** |  |
|  |  |  |  | Growth | r=0.16 |
|  |  |  |  | Communication | **r=0.52** |
|  |  |  |  | Stress | r=0.28 |
|  |  |  |  | Satisfaction | **r=0.29** |
|  |  |  |  | Mission | **r=0.49** |
|  |  |  | MCSS-26 formative sub-scale | **Organizational Readiness for Change** |  |
|  |  |  |  | Growth | r=0.12 |
|  |  |  |  | Communication | **r=0.53** |
|  |  |  |  | Stress | r=0.24 |
|  |  |  |  | Satisfaction | **r=0.31** |
|  |  |  |  | Mission | **r=0.49** |
|  |  |  | MCSS-26 normative sub-scale | **Organizational Readiness for Change** |  |
|  |  |  |  | Growth | r=0.25 |
|  |  |  |  | Communication | r=0.14 |
|  |  |  |  | Stress | r 0.14 |
|  |  |  |  | Satisfaction | r=0.20 |
|  |  |  |  | Mission | r=0.19 |

a – no measure of effect provided; b – outcome measure not validated; c – comparing high MCSS score (>141) to low MCSS score (≤141); d – comparing high MCSS-26 score (≥85) to low MCSS-26 score (<85); e – comparing high MCSS-26 score (≥75) to low MCSS-26 score (<75); f – comparing high MCSS-26 score (≥73) to low MCSS-26 score (<73).

MCSS score range 36 to 180; MCSS-26 score range 0 to 104.

Positive association for job retention, job satisfaction, perceptions of work environment and well-being indicates effectiveness of supervision is associated with better outcome.

Negative association for burnout indicates effectiveness of supervision is associated with better outcome.
