# Supplementary material for: Impact of clinical supervision on healthcare organisational outcomes: A mixed methods systematic review
Source: PLoS One. 2021 Nov 19;16(11):e0260156. doi: 10.1371/journal.pone.0260156 (PMC8604366; doi:10.1371/journal.pone.0260156)
Supplement: S8 Table — (DOCX) [file pone.0260156.s009.docx]

**Supplementary Table 8**. Results of studies investigating the association between an effective supervisor and organisational outcomes

| **Study** | **Design** | **n** | **Measure of perception of supervisor** | **Outcome** | **Result**  (Bold indicates statistical significance) |
| --- | --- | --- | --- | --- | --- |
| **Burnout** | | | | | |
| Ben-Porat  2011 | Cross sectional | 113 | Multifactorial leadership questionnaire | **Pines Burnout Questionnaire** | no association^a^ |
| Edwards  2006 | Cross sectional | 208 | MCSS | **Maslach Burnout Inventory** |  |
|  |  |  | Supervisor trust and rapport sub-scale | Emotional exhaustion | **r=-0.19** |
|  |  |  |  | Depersonalisation | **r=-0.23** |
|  |  |  |  | Personal accomplishment | no association^a^ |
|  |  |  | MCSS | **Maslach Burnout Inventory** |  |
|  |  |  | Supervisor advice and support sub-scale | Emotional exhaustion | no association^a^ |
|  |  |  |  | Depersonalisation | **r=-0.17** |
|  |  |  |  | Personal accomplishment | no association^a^ |
| Hyrkäs  2006 | Cross sectional | 542 | MCSS | **Maslach Burnout Inventory** |  |
|  |  |  | Supervisor trust and rapport sub-scale | Emotional exhaustion^c^ | N/S^a^ |
|  |  |  |  | Depersonalisation^c^ | N/S^a^ |
|  |  |  |  | Personal accomplishment^c^ | N/S^a^ |
|  |  |  | MCSS | **Maslach Burnout Inventory** |  |
|  |  |  | Supervisor advice and support sub-scale | Emotional exhaustion^d^ | **OR 1.53 (95%CI 1.03 to 2.89)** |
|  |  |  |  | Depersonalisation^d^ | N/S^a^ |
|  |  |  |  | Personal accomplishment^d^ | N/S^a^ |
| Livini  2012 | Cross sectional | 37 | Supervision Attitude Scale |  |  |
|  |  |  | (Individual Supervision Group) | **Maslach Burnout Inventory** | **r=-0.78** |
|  |  |  | (Group Supervision Group) | **Maslach Burnout Inventory** | no association^a^ |
| Webster  1999 | Cross sectional | 151 | Clinical Supervisor Rating Scale^b^ | **Maslach Burnout Inventory** |  |
|  |  |  |  | Emotional exhaustion | **r=-0.25** |
|  |  |  |  | Depersonalisation | **r=-0.22** |
|  |  |  |  | Personal accomplishment | r=-0.12 |
| **Job Satisfaction** | | | | | |
| Hyrkäs  2006 | Cross sectional | 542 | MCSS | **Minnesota Job Satisfaction Scale** |  |
|  |  |  | Supervisor trust and rapport sub-scale | Extrinsic job satisfaction^c^ | N/S^a^ |
|  |  |  |  | Intrinsic job satisfaction^c^ | **OR 1.56 (95%CI 1.08 to 2.27)** |
|  |  |  |  | Total job satisfaction^c^ | N/S^a^ |
|  |  |  | MCSS | **Minnesota Job Satisfaction Scale** |  |
|  |  |  | Supervisor advice and support sub-scale | Extrinsic job satisfaction^d^ | **OR 1.75 (95%CI 1.19 to 2.56)** |
|  |  |  |  | Intrinsic job satisfaction^d^ | N/S^a^ |
|  |  |  |  | Total job satisfaction^d^ | **OR 1.82 (95%CI 1.27 to 2.63)** |
| Livini  2012 | Cross sectional | 37 | Supervision Attitude Scale |  |  |
|  |  |  | (Individual Supervision Group) | **Intrinsic Job satisfaction Scale** | **r=0.58** |
|  |  |  | (Group Supervision Group) | **Intrinsic Job satisfaction Scale** | no association^a^ |
| Kavanagh 2003 | Cross sectional | 272 | Supervision Attitude Scale | **Hoppock Job Satisfaction Measure** | **r=0.30** |
|  |  |  |  |  | **β=0.09 (95%CI 0.02 to 0.17)** |
| Nathanson  1992 | Cross sectional | 193 | Satisfaction with Supervisor^b^ | **Job Satisfaction^b^** | **r=0.551** |
| Schroffel  1999 | Cross sectional | 84 | Perceived Supervisor’s Helpfulness^b^ | **Job Descriptive Index** |  |
|  |  |  |  | Work itself | **r=0.35** |
|  |  |  |  | Pay | no association^a^ |
|  |  |  |  | Promotional opportunities | no association^a^ |
|  |  |  |  | Co-workers | no association^a^ |
|  |  |  |  | Supervision | no association^a^ |
|  |  |  |  | **Job in General Index** | **r=0.32** |
| **Well-being** | | | | | |
| Ben-Porat  2011 | Cross sectional | 143 | Multifactorial leadership questionnaire | **Secondary Traumatic Stress Scale** | no association^a^ |
| Livini  2012 | Cross sectional | 37 | Supervision Attitude Scale |  |  |
|  |  |  | (Individual Supervision Group) | **Scales of Psychological Well-Being** | **r=0.60** |
|  |  |  | (Group Supervision Group) | **Scales of Psychological Well-Being** | no association^a^ |

a – no measure of effect provided; b – outcome measure not validated; c – comparing high MCSS score (≥30) to low MCSS score (<30); d – comparing high MCSS score (>23) to low MCSS score (≤23); N/S – no statistically significant difference between groups.

MCSS Supervisor Trust and Rapport range 7 to 35; MCSS Supervisor Advice and Support range 6 to 30.

Positive association for job satisfaction, role competence and well-being indicates perception of effective supervisor is associated with better outcome.

Negative association for burnout indicates perception of effective supervisor is associated with better outcome.
