# Supplementary material for: Impact of clinical supervision on healthcare organisational outcomes: A mixed methods systematic review
Source: PLoS One. 2021 Nov 19;16(11):e0260156. doi: 10.1371/journal.pone.0260156 (PMC8604366; doi:10.1371/journal.pone.0260156)
Supplement: S1 Appendix — (DOCX) [file pone.0260156.s010.docx]

**Appendix 1: Search Strategy**

**CINAHL**

|  | Search Terms |
| --- | --- |
| #1 | MH Clinical Supervision OR TIAB "clinical supervision" |
| #2 | TIAB "Organizational outcome" OR TIAB "organizational effectiveness" OR TIAB "staff turnover" OR MH personnel turnover OR TIAB "personnel turnover" OR TIAB "employee turnover" OR TIAB "staff retention" OR MH personnel retention OR TIAB "personnel retention" OR TIAB "employee retention" OR TIAB "staff recruitment" OR MH personnel recruitment OR TIAB "personnel recruitment" OR TIAB "employee recruitment" OR TIAB "retention and recruitment" OR TIAB "intent-to-stay" OR TIAB "intent-to-leave" OR MH Job Satisfaction OR TIAB "job satisfaction" OR MH Quality of Working Life OR TIAB "quality of work life" OR MH Absenteeism OR TIAB absenteeism OR MH Burnout, Professional OR TIAB "professional burnout" OR MH productivity OR TIAB productivity OR TIAB "organizational efficiency" |
| #3 | #1 AND #2 |

**EMBASE**

|  | Search Terms |
| --- | --- |
| #1 | 'clinical supervision'/exp OR 'clinical supervision' |
| #2 | 'Organizational outcome' OR 'organizational effectiveness' OR 'staff turnover'/exp OR 'staff turnover' OR 'personnel turnover' OR 'personnel turnover' OR 'employee turnover' OR 'staff retention' OR 'personnel retention' OR 'personnel retention' OR 'employee retention' OR 'staff recruitment' OR 'personnel recruitment' OR 'personnel recruitment' OR 'employee recruitment' OR 'retention and recruitment' OR 'intent-to-stay' OR 'intent-to-leave' OR 'job satisfaction'/exp OR 'job satisfaction' OR 'quality of working life'/exp OR 'quality of working life' OR 'absenteeism'/exp OR 'absenteeism' OR 'professional burnout'/exp OR 'professional burnout' OR 'productivity'/exp OR 'productivity' OR 'organizational efficiency'/exp OR 'organizational efficiency' |
| #3 | #1 AND #2 |

**PubMed**

|  | Search Terms |
| --- | --- |
| #1 | Clinical Supervis* [tiab] |
| #2 | Organizational outcome [tiab] OR organizational effectiveness[tiab] OR staff turnover[tiab] OR personnel turnover[tiab] OR employee turnover[tiab] OR staff retention[tiab] OR personnel retention[tiab] OR employee retention[tiab] OR staff recruitment[tiab] OR personnel recruitment[tiab] OR employee recruitment[tiab] OR retention and recruitment[tiab] OR intent-to-stay[tiab] OR intent-to-leave[tiab] OR Job Satisfaction[mh] OR job satisfaction[tiab] OR quality of work life[tiab] OR Absenteeism[mh] OR absenteeism[tiab] OR Burnout, Professional[mh] OR professional burnout[tiab] OR Efficiency, Organizational[mh] OR productivity[tiab] OR organizational efficiency[tiab] |
| #3 | #1 AND #2 |

**PsychInfo**

|  | Search Terms |
| --- | --- |
| #1 | clinical supervision.mp. or exp Professional Supervision/ |
| #2 | Organizational outcome.mp. OR organizational effectiveness.mp. OR staff turnover.mp. OR personnel turnover.mp. OR exp Employee Turnover/ OR staff retention.mp. OR personnel retention.mp. OR exp employee retention/ OR staff recruitment.mp. OR exp personnel recruitment/ OR employee recruitment.mp. OR retention and recruitment.mp. OR intent-to-stay.mp. OR intent-to-leave.mp. OR exp Job Satisfaction/ OR exp "Quality of Work Life"/ OR quality of work life.mp. OR exp employee absenteeism'/ OR absenteeism.mp. OR burnout.mp. OR productivity.mp. OR exp Employee Productivity/ OR organizational efficiency.mp. |
| #3 | #1 AND #2 |

**Scopus**

|  | Search Terms |
| --- | --- |
| #1 | TITLE-ABS-KEY ( "clinical supervision" ) |
| #2 | TITLE-ABS-KEY ( "Organizational outcome" ) OR TITLE-ABS-KEY ( "organizational effectiveness" ) OR TITLE-ABS-KEY ( "staff turnover" ) OR TITLE-ABS-KEY ( "personnel turnover" ) OR TITLE-ABS-KEY ( "Employee Turnover" ) OR TITLE-ABS-KEY ( "staff retention" ) OR TITLE-ABS-KEY ( "personnel retention" ) OR TITLE-ABS-KEY ( "employee retention" ) OR TITLE-ABS-KEY ( "staff recruitment" ) OR TITLE-ABS-KEY ( "personnel recruitment" ) OR TITLE-ABS-KEY ( "employee recruitment" ) OR TITLE-ABS-KEY ( "retention and recruitment" ) OR TITLE-ABS-KEY ( "intent-to-stay" ) OR TITLE-ABS-KEY ( "intent-to-leave" ) OR TITLE-ABS-KEY ( "Job Satisfaction" ) OR TITLE-ABS-KEY ( "Quality of Work Life" ) OR TITLE-ABS-KEY ( "employee absenteeism" ) OR TITLE-ABS-KEY ( absenteeism ) OR TITLE-ABS-KEY ( burnout ) OR TITLE-ABS-KEY ( productivity ) or TITLE-ABS-KEY ( "Employee Productivity" ) OR TITLE-ABS-KEY ( "organizational efficiency" ) |
| #3 | #1 AND #2 |
