# Supplementary material for: Impact of clinical supervision on healthcare organisational outcomes: A mixed methods systematic review
Source: PLoS One. 2021 Nov 19;16(11):e0260156. doi: 10.1371/journal.pone.0260156 (PMC8604366; doi:10.1371/journal.pone.0260156)
Supplement: S2 Appendix — (DOCX) [file pone.0260156.s011.docx]

**Appendix 2 – Excluded studies**

| **No** | **Reference** | **Reason for exclusion** |
| --- | --- | --- |
| 1 | Austin A, Gulema H, Belizan M…et al. 2015. Barriers to providing quality emergency obstetric care in Addis Ababa, Ethiopia: Healthcare providers' perspectives on training, referrals and supervision, a mixed methods study. *BMC Pregnancy and Childbirth*, 15(1): 10.1186/s12884-015-0493-4 | Outcome not of interest |
| 2 | Azar ST. 2000. Preventing burnout in professionals and paraprofessionals who work with child abuse and neglect cases: A cognitive behavioral approach to supervision. *Journal of Clinical Psychology,* 56(5): 643-663. <http://dx.doi.org/10.1002/%28SICI%291097-4679%28200005%2956:5%3C643::AID-JCLP6%3E3.0.CO;2-U> | Descriptive paper |
| 3 | Barron CC. 2019. A qualitative study of reflective supervision from the supervisee perspective: An ecological view. *Dissertation Abstracts International Section A: Humanities and Social Sciences*. 80(11-A{E}). | Wrong intervention |
| 4 | Bazant E, Sarkar S, Banda J…et al. 2014. Effects of a performance and quality improvement intervention on the work environment in HIV-related care: A quasi-experimental evaluation in Zambia. *Health and Quality of Life Outcomes*, 12(1)(73), <http://dx.doi.org/10.1186/1478-4491-12-73_old> | Outcome not of interest |
| 5 | Beitel M, Oberleitner L, Muthulingam D…et al. 2018. Experiences of burnout among drug counselors in a large opioid treatment program: A qualitative investigation. *Substance Abuse*, 39(2): 211-217. 10.1080/08897077.2018.1449051 | Wrong population |
| 6 | Berg SA. 2018. An exploration of the relationship between supervision and job satisfaction factors among residence directors and resident assistants. *Dissertation Abstracts International Section A: Humanities and Social Sciences*, 78(7-A{E}. | Wrong population |
| 7 | Bogo M, Paterson J, Tufford L & King R. 2011. Supporting front-line practitioners' professional development and job satisfaction in mental health and addiction. *Journal of Interprofessional Care*, 25(3): 209-214. 10.3109/13561820.2011.554240 | Outcome not of interest |
| 8 | Bono JE, Foldes HJ, Vinson G & Muros JP. 2007. Workplace emotions: The role of supervision and leadership. *Journal of Applied Psychology*, 92(5): 1357-1367. <http://dx.doi.org/10.1037/0021-9010.92.5.1357>. | Outcome not of interest |
| 9 | Brewer DP. 1995. The effects of supervisory support, counselor characteristics, the work setting, and social influences on burnout in mental health counsellors. *Dissertation Abstracts International: Section B: The Sciences and Engineering*, 55(9-B):4100, | Wrong population |
| 10 | Burnard P, Edwards D, Hannigan B…et al. 2003. The effectiveness of clinical supervision on burnout amongst community mental health nurses in Wales. *Journal of Psychiatric and Mental Health Nursing*, 10(2): 231-233. 10.1046/j.1365-2850.2003.00569.x | Descriptive paper |
| 11 | Carney S. 2005. Clinical supervision in a challenging behaviour unit. *Nursing times*, 101(47): 32-34. | Outcome not of interest |
| 12 | Caruso C, Costantini M & Mezzaluna C. The role of supervision in preventing burnout among psychotherapists: A pilot study. *Psicoterapia Cognitiva e Comportamentale*, 23(3): 317-330 | Wrong population |
| 13 | Choi J & Johantgen M. 2012. The importance of supervision in retention of CNAs. *Research in Nursing & Health*, 35(2):187-199. <http://dx.doi.org/10.1002/nur.21461> | Wrong population |
| 14 | Dickin KL, Dollahite JS & Habicht J. 2010. "Job satisfaction and retention of community nutrition educators: The importance of perceived value of the program, consultative supervision, and work relationships": Erratum. *Journal of Nutrition Education and Behavior*, 42(6): 429. <http://dx.doi.org/10.1016/j.jneb.2010.09.006>. | Descriptive paper |
| 15 | Circenis K & Millere I. 2012. Stress related work environment factors: Nurses survey results. *International Journal of Collaborative Research on Internal Medicine and Public Health*, 4(6):1150-1155. | Outcome not of interest |
| 16 | Cutcliffe J & McFeely S. 2001. Practice nurses and their 'lived experience' of clinical supervision. *British Journal of Nursing*, 10(5): 312-314, 316-323. | Outcome not of interest |
| 17 | Davis D. 2008. The ethics of self-care: Burnout among substance abuse professionals. *Dissertation Abstracts International: Section B: The Sciences and Engineering*, 69(1-B): 724. | Wrong population |
| 18 | Davis AH, Savicki V, Cooley EJ & Firth JL. 1989. Burnout and counselor practitioner expectations of supervision. *Counselor Education and Supervision*, 28(3): 234-241. <http://dx.doi.org/10.1002/j.1556-6978.1989.tb01112.x> | Wrong population |
| 19 | Deihl LM. 2009. The relevance of the supervisory working alliance to burnout and vigor among residential frontline staff. *Dissertation Abstracts International: Section B: The Sciences and Engineering*, 70(5-B): 3166. | Wrong population |
| 20 | Edwards JA, Kinsella J, Shaw A, Evans S & Anderson KJ. 2010. Sedation for oocyte retrieval using target controlled infusion of propofol and incremental alfentanil delivered by non-anaesthetists. *Anaesthesia*  , 65(5): 453-461. <http://dx.doi.org/10.1111/j.1365-2044.2010.06264.x>. | Wrong intervention |
| 21 | Elmcrona M & Winroth MK. 1997. Clinical supervision--experience of 10 nurses of a 2-year process-oriented supervision. *Vård i Norden*, 17(3): 4-9. | Not in English |
| 22 | Evans WN & Hohenshil TH. 1997. Job Satisfaction of Substance Abuse Counselors. *Alcoholism Treatment Quarterly*, 15(2): 1-13. 10.1300/J020v15n02_01 | Wrong population |
| 23 | Franco GE. 2015. Supervision and MFT burnout: Overcoming the challenges therapists face in the workplace. *Frontiers in Psychology,* 6. 10.3389/fpsyg.2015.01644. | Descriptive paper |
| 24 | Frosh CA, Varwani Z, Mitchell J, Caraccioli C & Willoughby M. 2018. Impact of reflective supervision one arly childhood interventionists’ perceptions of self-efficacy, job satisfaction and job stress. *Infant Mental Health Journal*, 39(4): 385-395. 10.1002/imhj.21718. | Wrong population |
| 25 | Fukuyama K. 1999. Influences of selected characteristics of professional supervision on job satisfaction, productivity and autonomy of professional social workers in Japan. *Dissertation Abstracts International Section A: Humanities and Social Sciences*, 60 (4-A): 1328. | Outcome not of interest |
| 26 | Gachutha CW. 2009. The role of supervision in the management of counsellor burnout. *Dissertation Abstracts International: Section B: The Sciences and Engineering*, 69(11-B): 7138. | Wrong population |
| 27 | Hallberg IR & Norberg A. 1993. Strain among nurses and their emotional reactions during 1 year of systematic clinical supervision combined with the implementation of individualized care in dementia nursing. *Journal of Advanced Nursing*, 18(12):1860-1875. 10.1046/j.1365-2648.1993.18121860.x | Outcome not of interest |
| 28 | Jack H, Canavan M, Ofori-Atta A, Taylor L & Bradley E. 2013. Recruitment and Retention of Mental Health Workers in Ghana. *PLoS ONE*, 8(2). <http://dx.doi.org/10.1371/journal.pone.0057940>. | Outcome not of interest |
| 29 | Jerell JM. 1983. Work satisfaction among rural mental health staff. *Community Mental Health Journal*, 19(3): 187-200. <http://dx.doi.org/10.1007/BF00759552> | Wrong intervention |
| 30 | Knudsen HK, Ducharme LJ & Roman PM. 2008. Clinical supervision, emotional exhaustion, and turnover intention: A study of substance abuse treatment counselors in the Clinical Trials Network of the National Institute on Drug Abuse. *Journal of Substance Abuse Treatment*,35(4): 387-395. <http://dx.doi.org/10.1080/07325223.2013.850139> | Wrong population |
| 31 | Knudsen HK, Roman PM & Abraham AJ. 2013. Quality of clinical supervision and counselor emotional exhaustion: The potential mediating roles of organizational and occupational commitment. *Journal of Substance Abuse Treatment*, 44(5): 528-533. 10.1016/j.jsat.2012.12.003. | Outcome not of interest |
| 32 | Koob JJ. 2003. The effects of Solution-Focused supervision on the perceived Self-Efficacy of therapists in training. *Clinical Supervisor,* 21(2): 161-183. 10.1300/J001v21n02_11 | Wrong population |
| 33 | Lynch L & Heppell B. 2008. Implementing clinical supervision: Part 1: laying the groundwork. *International Journal of Mental Health Nursing*. 2008 Feb;17(1):57-64. doi: 10.1111/j.1447-0349.2007.00511.x. | Outcome not of interest |
| 34 | Mackereth PA, Parkin S, Donald G & Antcliffe N. 2010. Clinical supervision and complementary therapists: An exploration of the rewards and challenges of cancer care. *Complementary Therapies in Clinical Practice*, 16(3):143-148. 10.1016/j.ctcp.2010.01.001. | Wrong population |
| 35 | MacLaren J, Stenhouse R & Ritchie D. 2016. Mental health nurses' experiences of managing work-related emotions through supervision. *Journal of Advanced Nursing*, 72(10): 2423-2434. 10.1111/jan.12995 | Outcome not of interest |
| 36 | Marrow CE, Macauley DM & Crumbie A. 1997. Promoting reflective practice through structured clinical supervision. *Journal of Nursing Management,* 5(2): 77-82. | Outcome not of interest |
| 37 | McGarry J, Aubeeluck A & De Oliveira D. 2019. Evaluation of an evidence-based model of safeguarding clinical supervision within one healthcare organization in the United Kingdom. *International Journal of Evidence-based Healthcare,* 17(s1): s29-s31. <http://dx.doi.org/10.1097/XEB.0000000000000180>. | Outcome not of interest |
| 38 | McNamara J & Gillies RM. 2003. Support Requirements and Effects of Supervision on Telephone Counsellors. *Australian Journal of Guidance and Counselling*, 13(2): 192-204. <http://dx.doi.org/10.1017/S1037291100002880>. | Wrong population |
| 39 | Severinsson EI. 1995. The phenomenon of clinical supervision in psychiatric health care. *Journal of Psychiatric and Mental Health Nursing*, 2(5): 301-309. | Outcome not of interest |
| 40 | Severinsson E & Sand A. 2010. Evaluation of the clinical supervision and professional development of student nurses. *Journal of Nursing Management*, 18(6): 669-677. | Wrong population |
| 41 | Sloan G & Watson H. 2001. Illuminative evaluation: evaluating clinical supervision on its performance rather than the applause. *Journal of Advanced Nursing*, 35(5): 664-673. | Outcome not of interest |
| 42 | Starr F, Ciclitira K, Brunswick N & Costa A. 2013. Comfort and challenge: a thematic analysis of female clinicians' experiences of supervision. *Psychology and Psychotherapy*, 86(3): 334-351. | Outcome not of interest |
| 43 | Sterner WR. 2007. The influence of the supervisory working alliance on work satisfaction and work-related stress for counselors in professional settings. *Dissertation Abstracts International Section A: Humanities and Social Sciences*, 68(5-A): 1833 | Wrong population |
| 44 | Sterner WR. 2009. Influence of the supervisory working alliance on supervisee work satisfaction and work-related stress. *Journal of Mental Health Counseling*, 31(3): 249-263. <http://dx.doi.org/10.17744/mehc.31.3.f3544l502401831g> | Wrong population |
| 45 | Taylor M & Harrison CA. 2010. Introducing clinical supervision across Western Australian public mental health services. *International Journal of Mental Health Nursing*, 19(4): 287-293. 10.1111/j.1447-0349.2010.00675.x | Outcome not of interest |
| 46 | Thielking M, Susan M & Jimerson SR. 2006. Supervision and Satisfaction Among School Psychologists: An Empirical Study of Professionals in Victoria, Australia. *School Psychology*, 27(4): 405-414. <http://dx.doi.org/10.1177/0143034306070426> | Wrong setting |
| 47 | Thornberry K, Roeland E & Mitchell B. 2014. Clinical supervision: An antidote to personal and team compassion fatigue. *Journal of Pain and Symptom Management*, 47(2): 447-448. <http://dx.doi.org/10.1016/j.jpainsymman.2013.12.199>. | Descriptive paper |
| 48 | Vermilyea EG. 2015. Trauma-informed training and clinical supervision as moderators of compassion fatigue, when controlling for burnout and a personal history of trauma. *Dissertation Abstracts International: Section B: The Sciences and Engineering*, 75(10-B[E]). | Outcome not of interest |
| 49 | Wallbank S & Woods G. 2012. A healthier health visiting workforce: Findings from the restorative supervision programme. *Community Practitioner*, 85(11): 20-23. | Wrong intervention |
| 50 | Walsh-Rock VJ. 2018. Exploring the clinical supervision experiences of school counselors: Perceptions of counseling skills and professional identity. *Dissertation Abstracts International Section A: Humanities and Social Sciences*, 79(10-A[E]). | Wrong population |
| 51 | Weigelt J. 2016. Supervisory working alliance and job satisfaction in community mental health settings. *Dissertation Abstracts International: Section B: The Sciences and Engineering*, 77 (2-B[E]) | Wrong population |
| 52 | White AD. 2018. Helping the helper: Analyzing the effects of clinical supervision on levels of burnout. *Dissertation Abstracts International Section A: Humanities and Social Sciences*, 79 (1-A[E]). | Wrong population |
| 53 | White E & Winstanley J. 2006. Cost and resource implications of clinical supervision in nursing: An Australian perspective. *Journal of Nursing Management*, 14(8): 628-636. 10.1111/j.1365-2934.2006.00721.x. | Outcome not of interest |

**Duplicate articles eliminated prior to the full text screening stage:**

1. Hallberg IR, Hansson UW, Axelsson K. Satisfaction with nursing care and work during a year of clinical supervision and individualize care. Comparison between two wards for the care of severly demented patients. Journal of Nursing Management 1994;1:297-307.
2. Hyrkäs K, Appelqvist-Schmidlechner K, Haataja R. Efficacy of clinical supervision: influence on job satisfaction, burnout and quality of care. Journal of Advanced Nursing 2006;55(4):521-535.
3. Koivu A, Saarinen PI, Hyrkäs K. Does clinical supervision promote medical-surgical nurses’ well-being at work? A quasi-experimental 4-year follow-up study. Journal of Nursing Management 2012;20:401-413.
