# Supplementary material for: Impact of clinical supervision on healthcare organisational outcomes: A mixed methods systematic review
Source: PLoS One. 2021 Nov 19;16(11):e0260156. doi: 10.1371/journal.pone.0260156 (PMC8604366; doi:10.1371/journal.pone.0260156)
Supplement: Attachment — Submitted filename: PONE-D-21-20050_reviewer.pdf [file pone.0260156.s012.pdf]

PLOS ONE

Impact of clinical supervision on healthcare organisational outcomes: a mixed methods systematic review

--Manuscript Draft--

**Manuscript Number:**	PONE-D-21-20050
**Article Type:**	Research Article
**Full Title:**	Impact of clinical supervision on healthcare organisational outcomes: a mixed methods systematic review
**Short Title:**	Impact of clinical supervision on healthcare organisational outcomes
**Corresponding Author:**	Priya Martin<br>UQ: The University of Queensland<br>Toowoomba, QLD AUSTRALIA
**Keywords:**	Clinical supervision; organisational outcomes; healthcare professionals
**Abstract:**	Objective<br>To review the impact of clinical supervision of post-registration/qualification healthcare professionals on healthcare organisational outcomes.<br>Background<br>Clinical supervision is a professional support mechanism that benefits patients, healthcare professionals and healthcare organisations. Whilst evidence is growing on the impact of clinical supervision on patient and healthcare professional outcomes, the evidence base for the impact of clinical supervision on organisational outcomes remains weak.<br>Methods<br>This review used a convergent segregated approach to synthesise and integrate quantitative and qualitative research findings, as per the Joanna Briggs Institute's recommendations for mixed methods systematic reviews. Databases searched included CINAHL, Embase, PubMed, PschINFO, and Scopus. Whilst a narrative synthesis was performed to present the findings of the quantitative and qualitative studies, the evidence from both quantitative and qualitative studies was subsequently integrated for a combined presentation. The review followed the guidelines of the Preferred Reporting Items for Systematic Reviews and Meta-Analyses.<br>Results<br>Thirty-two studies including 27 quantitative, two qualitative and three mixed methods studies, were included in the review. The results of the quantitative analysis showed that effective clinical supervision was associated with lower burnout and greater staff retention, and effective supervisor was associated with lower burnout and greater job satisfaction. Qualitative findings showed that healthcare professionals believed that adequate clinical supervision could mitigate the risk of burnout, facilitate staff retention, and improve the work environment, while inadequate clinical supervision can lead to stress and burnout. The evidence from quantitative and qualitative studies were complementary of each other.<br>Conclusion<br>Clinical supervision can have a variable effect on healthcare organisational outcomes. The direction of this effect appears to be influenced by the effectiveness of both the clinical supervision provided and that of the clinical supervisor. This highlights the need for organisations to invest in high quality supervision practices if maximal gains from clinical supervision are to be attained.
**Order of Authors:**	Priya Martin
	Lucylynn Lizarondo
	Saravana Kumar
	David Snowdon
**Additional Information:**	
**Question**	**Response**

Powered by Editorial Manager® and ProduXion Manager® from Aries Systems Corporation

**Financial Disclosure**<br><br>Enter a financial disclosure statement that describes the sources of funding for the work included in this submission. Review the submission guidelines for detailed requirements. View published research articles from *PLOS ONE* for specific examples.<br><br>This statement is required for submission and **will appear in the published article** if the submission is accepted. Please make sure it is accurate.<br><br>**Unfunded studies**<br>Enter: *The author(s) received no specific funding for this work.*<br><br>**Funded studies**<br>Enter a statement with the following details:<br>• Initials of the authors who received each award<br>• Grant numbers awarded to each author<br>• The full name of each funder<br>• URL of each funder website<br>• Did the sponsors or funders play any role in the study design, data collection and analysis, decision to publish, or preparation of the manuscript?<br>• **NO** - Include this sentence at the end of your statement: *The funders had no role in study design, data collection and analysis, decision to publish, or preparation of the manuscript.*<br>• **YES** - Specify the role(s) played.<br><br>* typeset	The authors received no specific funding for this work
**Competing Interests**<br><br>Use the instructions below to enter a competing interest statement for this submission. On behalf of all authors, disclose any competing interests that could be perceived to bias this work—acknowledging all financial support and any other relevant financial or non-financial competing interests.<br><br>This statement is required for submission	The authors have declared that no competing interests exist

and **will appear in the published article** if the submission is accepted. Please make sure it is accurate and that any funding sources listed in your Funding Information later in the submission form are also declared in your Financial Disclosure statement.<br><br>View published research articles from *PLOS ONE* for specific examples.<br><br>**NO authors have competing interests**<br><br>Enter: *The authors have declared that no competing interests exist.*<br><br>**Authors with competing interests**<br><br>Enter competing interest details beginning with this statement:<br><br>*I have read the journal's policy and the authors of this manuscript have the following competing interests: [insert competing interests here]*<br><br>* typeset	
**Ethics Statement**<br><br>Enter an ethics statement for this submission. This statement is required if the study involved:<br><br>• Human participants<br>• Human specimens or tissue<br>• Vertebrate animals or cephalopods<br>• Vertebrate embryos or tissues<br>• Field research<br><br>Write "N/A" if the submission does not require an ethics statement.<br><br>General guidance is provided below. Consult the submission guidelines for detailed instructions. **Make sure that all information entered here is included in the Methods section of the manuscript.**	N/A

**Format for specific study types**<br><br>**Human Subject Research (involving human participants and/or tissue)**<br>• Give the name of the institutional review board or ethics committee that approved the study<br>• Include the approval number and/or a statement indicating approval of this research<br>• Indicate the form of consent obtained (written/oral) or the reason that consent was not obtained (e.g. the data were analyzed anonymously)<br><br>**Animal Research (involving vertebrate animals, embryos or tissues)**<br>• Provide the name of the Institutional Animal Care and Use Committee (IACUC) or other relevant ethics board that reviewed the study protocol, and indicate whether they approved this research or granted a formal waiver of ethical approval<br>• Include an approval number if one was obtained<br>• If the study involved *non-human primates*, add *additional details* about animal welfare and steps taken to ameliorate suffering<br>• If anesthesia, euthanasia, or any kind of animal sacrifice is part of the study, include briefly which substances and/or methods were applied<br><br>**Field Research**<br><br>Include the following details if this study involves the collection of plant, animal, or other materials from a natural setting:<br>• Field permit number<br>• Name of the institution or relevant body that granted permission	
**Data Availability**<br><br>Authors are required to make all data underlying the findings described fully available, without restriction, and from the time of publication. PLOS allows rare exceptions to address legal and ethical concerns. See the PLOS Data Policy and FAQ for detailed information.	Yes - all data are fully available without restriction

A Data Availability Statement describing where the data can be found is required at submission. Your answers to this question constitute the Data Availability Statement and **will be published in the article**, if accepted.<br><br>**Important:** Stating 'data available on request from the author' is not sufficient. If your data are only available upon request, select 'No' for the first question and explain your exceptional situation in the text box.<br><br>Do the authors confirm that all data underlying the findings described in their manuscript are fully available without restriction?	
**Describe where the data may be found in full sentences. If you are copying our sample text, replace any instances of XXX with the appropriate details.**<br><br>• If the data are **held or will be held in a public repository**, include URLs, accession numbers or DOIs. If this information will only be available after acceptance, indicate this by ticking the box below. For example: *All XXX files are available from the XXX database (accession number(s) XXX, XXX.)*.<br>• If the data are all contained **within the manuscript and/or Supporting Information files**, enter the following: *All relevant data are within the manuscript and its Supporting Information files.*<br>• If neither of these applies but you are able to provide **details of access elsewhere**, with or without limitations, please do so. For example:<br><br>*Data cannot be shared publicly because of [XXX]. Data are available from the XXX Institutional Data Access / Ethics Committee (contact via XXX) for researchers who meet the criteria for access to confidential data.*<br><br>*The data underlying the results presented in the study are available from (include the name of the third party*	All relevant data are within the manuscript and its Supporting Information files.

and contact information or URL).<br>• This text is appropriate if the data are owned by a third party and authors do not have permission to share the data.<br><br>* typeset	
Additional data availability information:	

Manuscript
Click here to access/download;Manuscript;Manuscript_FNL_17Jun21.docx

Title Page

Title - Impact of clinical supervision on healthcare organisational outcomes: a mixed methods systematic review

Author information

1. Dr Priya Martin (Corresponding author)
Affiliation One:
Senior Research Fellow
Rural Clinical School, Faculty of Medicine,
The University of Queensland
Toowoomba, QLD- 4350
Australia

Affiliation Two:
Advanced Clinical Educator -Interprofessional
Advance Queensland Industry Research Fellow
Cunningham Centre
Darling Downs Health
Toowoomba, QLD-4350
Australia

Email – Priya.Martin@uq.edu.au
Ph - +61 7 4699 8126

2. Dr Lucylynn Lizarondo
Research Fellow: Implementation Science
Joanna Briggs Institute
The University of Adelaide
Adelaide, SA – 5005
Australia
Email – Lucylynn.Lizarondo@adelaide.edu.au

3. A/Prof Saravana Kumar
Allied Health and Human Performance
University of South Australia
Adelaide, SA – 5000
Australia

Email - Saravana.Kumar@unisa.edu.au

4. Dr David Snowdon
Affiliation One:
Research Fellow
Peninsula Clinical School
Central Clinical School
Monash University
Melbourne, VIC – 3199
Australia

Affiliation Two:
Allied Health Research Lead
Academic Unit
Peninsula Health
Melbourne, VIC – 3199
Australia
Email - David.Snowdon@monash.edu

Abstract

Objective

To review the impact of clinical supervision of post-registration/qualification healthcare professionals on healthcare organisational outcomes.

Background

Clinical supervision is a professional support mechanism that benefits patients, healthcare professionals and healthcare organisations. Whilst evidence is growing on the impact of clinical supervision on patient and healthcare professional outcomes, the evidence base for the impact of clinical supervision on organisational outcomes remains weak.

Methods

This review used a convergent segregated approach to synthesise and integrate quantitative and qualitative research findings, as per the Joanna Briggs Institute's recommendations for mixed methods systematic reviews. Databases searched included CINAHL, Embase, PubMed, PschINFO, and Scopus. Whilst a narrative synthesis was performed to present the findings of the quantitative and qualitative studies, the evidence from both quantitative and qualitative studies was subsequently integrated for a combined presentation. The review followed the guidelines of the Preferred Reporting Items for Systematic Reviews and Meta-Analyses.

Results

Thirty-two studies including 27 quantitative, two qualitative and three mixed methods studies, were included in the review. The results of the quantitative analysis showed that effective clinical supervision was associated with lower burnout and greater staff retention, and effective supervisor was associated with lower burnout and greater job satisfaction. Qualitative findings showed that healthcare professionals believed that adequate clinical supervision could mitigate the risk of burnout, facilitate staff retention, and improve the work environment, while inadequate clinical supervision can lead to stress and burnout. The evidence from quantitative and qualitative studies were complementary of each other.

Conclusion

Clinical supervision can have a variable effect on healthcare organisational outcomes. The direction of this effect appears to be influenced by the effectiveness of both the clinical supervision provided and that of the clinical supervisor. This highlights the need for organisations to invest in high quality supervision practices if maximal gains from clinical supervision are to be attained.

Introduction

Clinical supervision is widely practiced in health and social care professions across the globe owing to its beneficial effects to patients, health professionals and organisations. Operationally, clinical supervision is viewed as a process that provides quarantined time and an opportunity to further develop the supervisee's reflective skills, within the context of an ongoing professional relationship, usually with an experienced practitioner (one-to-one supervision), or with peers (peer group supervision). The aim of clinical supervision is for the supervisee to engage in guided reflection on current practice in ways designed to develop and enhance that practice in the future [1,2]. This type of supervision involves reflective thinking, and discussion regarding professional development issues, caseload, clinical issues, and staff interpersonal issues. Issues in clinical supervision definition and terminologies are widely prevalent [2]. In this review, the following definition of clinical supervision has been adopted:

> "*The formal provision, by approved supervisors, of relationship-based education and training that is work-focused, and which manages, supports, develops and evaluates the work of colleague/s*" [1].

Whilst efforts are growing to strengthen the evidence in this area, there is also criticism about a vast majority of evidence on supervision, as being *proof by association* or tentative [3]. While there is a growing evidence base for the impact of clinical supervision on patient outcomes such as reduced risk of mortality, reduced risk of complications and more effective care [4-7], and health professional outcomes such as being better supported in their roles [8], there remains a

need to systematically review the evidence for the impact of clinical supervision on organisational outcomes, to further strengthen the evidence base on clinical supervision.

Determining the impact of clinical supervision on healthcare organisations, however, is difficult given the challenges in defining organisational outcomes and the overlapping nature of patient, health professional and organisational outcomes. For example, improved patient outcomes (e.g. improved morbidity and mortality) can satisfy multiple targets for ~~the~~ healthcare organisations, as can health professional outcomes (e.g. reduction in stress and burnout), which can reduce staff sick leave, a usual key performance indicator for organisations. In determining the organisational outcomes of interest for this review, we undertook a scan of the broader literature. A recent systematic review of leadership styles and outcome patterns for the nursing workforce and work environment, grouped the outcomes into six categories: staff satisfaction and job factors, staff relationships with work, staff health and wellbeing, relations among staff, organisational environment factors and productivity and effectiveness [9]. Another systematic review on the relationship between governance mechanisms in healthcare and health workforce outcomes considered staff turnover and job satisfaction [10]. Other organisational outcomes cited in the clinical supervision literature include improved teamwork [11] and job satisfaction [12]. In considering all this, organisational outcomes in this review will reflect the well-being of health professionals resulting from clinical supervision, that lead to better outcomes for the organisations such as recruitment and retention, intent-to-stay, intent-to-leave, job satisfaction and quality of work life, burnout and absenteeism. Furthermore, to date,

no review has explored health professionals' perspectives of, and the impact from, clinical supervision on organisational outcomes.

Therefore, as means of addressing these knowledge gaps, using a mixed methods design, this review aims to answer the following research questions:

1) What are the effects of clinical supervision of healthcare professionals on organisational outcomes?
2) What are healthcare professionals' experiences, views, and opinions regarding clinical supervision as it relates to organisational processes and outcomes?
3) What can be inferred from the qualitative synthesis of healthcare professionals' experiences/ views that can explain the effects of clinical supervision or inform its appropriateness and acceptability for health professionals?

Methods

This systematic review was conducted using Joanna Briggs Institute (JBI) methodology for mixed methods systematic review, specifically the convergent segregated approach to synthesis and integration [13]. The review followed the Preferred Reporting Items for Systematic Reviews and Meta-Analyses (PRISMA) guideline [14] and was based on an a-priori published protocol [15].

Inclusion criteria

The review protocol indicated the inclusion of studies that focused on one-to-one clinical supervision rather than group supervision. However, during the screening of studies, it became

apparent that there was a prevalence of studies that investigated both one-to-one and group supervision, and studies that did not specify the type of clinical supervision investigated. Given this challenge, and to reflect the reality of healthcare organisations utilising both these types of supervision regularly, the review team agreed to include any study on clinical supervision, regardless of the type (i.e. one-to-one or group). To be eligible, studies had to meet the following criteria: (1) investigated clinical supervision of qualified or registered health professionals (i.e. clinical supervision of post-qualified/registered health professionals, where they engage in one-to-one or group supervision sessions that happen over a period of time); (2) used qualitative, quantitative or mixed-methods study design; (3) if a quantitative study, examined the effects of clinical supervision on organisational outcomes, such as staff retention and recruitment, intent to stay, intent to leave, job satisfaction and quality of work life, burnout, and absenteeism; (4) if a qualitative study, explored health professionals' experiences, views, or opinions regarding clinical supervision as they relate to organisational outcomes.

Search strategy

As means of avoiding publication and location bias, the search strategy was developed to identify black (commercially published) and grey literature. An initial limited search of PubMed and CINAHL was undertaken followed by analysis of text words contained in the title and abstract and the index terms used to describe the articles. The search strategy, including all identified keywords and index terms, was then adapted for each database. The search for published studies was performed from the date of inception until May 2020 in the following databases: CINAHL, Embase, PubMed, PsycINFO, and Scopus. These databases were chosen as

they commonly include literature from health disciplines, a combination of discipline specific (e.g. CINAHL includes nursing and allied health literature) and multi-disciplinary (e.g. Scopus) and are routinely used in systematic reviews. The search for grey literature was undertaken in ProQuest Dissertations and Theses, Google Scholar and WorldWideScience.org. Pearling was also used to identify additional publications. The search strategy for each database is shown in Appendix 1.

Study selection

Following the search, all identified citations were collated and uploaded into EndNote X8.2 (Clarivate Analytics, PA, USA) [16] and duplicates removed. Two reviewers independently screened the titles and abstracts (LL and DS) against the inclusion criteria for the review. Potentially relevant articles were retrieved in full and assessed independently for eligibility by two other reviewers (PM and SK). Disagreements were resolved through discussion and consensus. Studies that did not meet the inclusion criteria were excluded and reasons for their exclusion are provided in Appendix 2. The PRISMA flow diagram of included studies is available in Figure 1.

[Insert Figure 1 about here].

Quality assessment

All eligible studies were assessed for methodological quality by two independent reviewers (PM and DS for quantitative studies; PM and LL for qualitative studies using the relevant JBI critical

appraisal tools [17]. These tools were chosen as they assist in assessing the trustworthiness, relevance and results of published studies and are widely used. Any disagreements that arose between the reviewers were resolved through discussion. All studies, regardless of the results of their methodological quality, underwent data extraction and synthesis.

Data extraction

For the quantitative component, data were extracted from quantitative and mixed methods studies (quantitative component only) and included specific details about the supervisee and supervisor characteristics (sample size, profession), characteristics of the supervision (type, frequency, duration), study design, setting, clinical supervision characteristics, outcomes measured, and results related to the organisational outcomes. For the qualitative component, data were extracted from qualitative and mixed methods studies (qualitative component only) and included specific details about the supervisee and supervisor characteristics (sample size, profession, work experience), study design and methods, setting, and findings which included participants' experiences of clinical supervision as they relate to organisational outcomes. Findings extracted from individual studies consisted of themes or subthemes reported by the authors. These findings were accompanied by a direct quotation representing a participant's voice (i.e. illustration). Findings were also assigned one of three levels of credibility according to the following criteria: (1) unequivocal: findings accompanied by an illustration that is beyond reasonable doubt and therefore not open to challenge, (2) credible: findings accompanied by an illustration lacking clear association with it and therefore open to challenge, and (3) not supported: findings are not supported by data. The review team discussed the data extraction

process, established standards and consistencies on how this should occur, and those with quantitative expertise (DS) and qualitative expertise (LL) lead the extraction process, with the primary reviewer (PM) acting as the additional reviewer for validation purposes.

Data synthesis and integration

A convergent segregated approach to synthesis and integration was applied [13]. This involved an initial independent synthesis of the quantitative studies and qualitative studies followed by the integration of findings from such syntheses using configurative analysis.

Quantitative data were analysed descriptively; meta-analyses were deemed not appropriate due to heterogeneity between studies in terms of clinical supervision interventions and participants. Odds ratios (OR) of dichotomous events and standardised mean differences (SMD) for continuous measures were calculated. For experimental studies OR were converted to SMD using an online calculator [18], to assist with interpretation of effect size. For observational analytical studies the correlational coefficient (r) was calculated in addition to OR and SMD. Effect size was determined using the following reference values for SMD: small 0.2, medium 0.5, large 0.8 [19]; OR: small 1.68, medium 3.47, large 6.71 [20]; and r: small 0.1, moderate 0.3, large 0.5 [19].

Qualitative synthesis was conducted using the meta-aggregative approach [21]. This involved assembling and aggregating the extracted findings from individual studies, based on similarity in meaning, to generate a set of statements (i.e. categories) that represented that aggregation.

These categories were then subjected to meta-synthesis to produce a set of synthesised findings. The findings of each single method synthesis were juxtaposed and examined for convergence/divergence and complementarity. To explore the relationship across individual syntheses, the findings were reviewed to determine whether they were supportive or contradictory and identify which aspects of the quantitative evidence were not explored in the qualitative studies and vice-versa. The clinical supervision interventions which had been investigated in the quantitative studies were further analysed in line with the experiences of participants in the qualitative studies to explain the impact of clinical supervision on the different organisational outcomes. However, due to the heterogeneity of the quantitative studies and the lack of well-conducted trials, and the limited qualitative studies, no clear cause and effect relationships can be determined, nor in-depth analysis can be made to explain the impact of clinical supervision.

Results

The database search yielded 1266 records. Eighty-five articles were retrieved for full text review following application of the eligibility criteria to title and abstract. Thirty-four fulfilled the inclusion criteria when applied to full texts. Three of these articles were duplicate publications, resulting in a yield of 31 studies. One article was identified through pearling of references in the included studies; hence the final yield was 32 studies (Figure 1).

Study characteristics

Twenty-seven quantitative [22-48], two qualitative [49-50] and three mixed methods studies [51-53] were included in the review. Fifteen studies used a randomised controlled (n=1) [22] or quasi-experimental design (n=14) [23-35, 51] to establish the effect of clinical supervision on organisational outcomes. Eight studies investigated the association between effective clinical supervision and organisational outcomes [37-43, 52]. Eight studies investigated the association between an effective supervisor and organisational outcomes [32, 36, 39, 41, 43, 45, 46, 48]. Two studies used a cross sectional survey study design to measure perceptions of effect of clinical supervision on organisational outcomes [44,47]. Four studies [49-52] used a qualitative descriptive design, with either individual [49-51] or focus group [52], semi structured interviews as the method of data collection. The qualitative component of one study [53] applied the grounded theory methodology, using a qualitative questionnaire for data collection.

Studies were most commonly conducted in the mental health setting (n=15) [25-28, 30, 34, 37, 39, 42-44, 46-48, 53] with nursing (n=23) [22-26, 30-35, 37,39,40-42,44,47, 48, 51, 53] and social work/psychology/counselling professionals (n=9) [22, 27, 32, 36, 38, 43, 45, 46, 48]. One study included various settings such as community, hospital, residential care, and general practice, with nursing staff as participants [49]. Another study focused on rural and regional supervision involving allied health professionals [50]. Seven studies were conducted in Sweden, [23,25,26,28,30,34,47] seven in Australia [32,38,40,43,50-52], seven in the United Kingdom [22, 35,37,39,44,49,53], four in the United States of America [27,45, 46,48], two in Finland [31,41] and one each in Norway [24], Israel [36], Africa [33], Denmark [42] and Italy [29]. Eight studies investigated only group supervision [23-26,30,31,42,47] three studies investigated only individual (one-to-one) supervision [22,27,38], 11 studies investigated both group and

individual supervision [29,32,35,39,41,43-46,51,52], one study investigated multi-professional supervision [28] and seven studies did not state the type of supervision investigated [33,34,36,37,40,48,53]. The frequency and duration of supervision sessions were variable between studies, ranging from weekly to every three months, and 30 to 480 minutes.

Five studies (two qualitative [49,50] and three mixed methods studies [51-53]) explored the clinical supervision experiences of healthcare professionals including its impact on clinical practice. Fifteen studies investigated the effect of supervision on burnout [22,25,27-32,35-37,39,41,42,48,52], 9 studies on other measures of well-being [22,24-26,30-32,42,44], 13 studies on job satisfaction [25-28,30,32,33,41-43,45,46,51], 9 studies on the work environment [23-26,31,34,35,38,47], and 3 studies on job retention [32,33,40]. There was a large diversity of outcome measures used with only four measures used in more than one study; the Maslach Burnout Inventory was used in 13 studies [25,27,29-32,35,37,39,41,42,48,52], and the Creative Climate Questionnaire [25,26], Tedium Measure [25,30] and Satisfaction with Nursing Care questionnaire [25,30] each used in two studies. Study characteristics can be found in table 1.
[Insert Table 1 about here].

Methodological quality

The predominant methodological risk of bias for analytical cross-sectional cohort studies (n=14) was the absence of strategies to deal with confounding factors [36,39,41,45,46,48,52]. For quasi-experimental studies (n=14) it was unclear if participants received similar support interventions other than clinical supervision in 12 studies [23-29,31,33-35,51], outcome measurement was not performed both pre and post intervention (i.e. multiple time points) in

nine studies [24,27-29,31,33-35,51], and it was unclear if participants were similar at baseline in seven studies [24,27-29,33,34,51]. The single randomised controlled trial [22] only met five of the 13 items; notably the method of randomisation was unclear and there was no between group statistical comparison. JBI Critical Appraisal Checklists can be found in supplementary table 1, 2 and 3.

The methodological quality of the five qualitative studies (including the qualitative component of mixed methods studies) was generally high. Two studies [51,52] scored 10 out of 10, while two other studies [49,50] scored eight out of 10, failing to account for the potential influence of the researcher on the research findings. One study [53] did not demonstrate congruity between their stated philosophical perspective and the research methodology used, nor was there congruence between their research methodology and their research question/objectives, methods of data collection and analysis and interpretation of results. The JBI Critical Appraisal Checklist can be found in supplementary table 4.

Impact of clinical supervision on organisational outcomes (Quantitative findings)

1. Clinical supervision compared to control

Eleven studies evaluated the effect of clinical supervision on organisational outcomes by comparison to a control group that did not receive clinical supervision [22,24,25,27-29,31,33-35,51]. While individual studies found clinical supervision had a positive effect on organisational outcomes, there were variable results across studies for burnout (figure 2A, 2B, 2C, 2D), job satisfaction, well-being, and workplace environment. Notably, a single randomised controlled

trial found that clinical supervision had a large effect on burnout (Figure 2D) and well-being [22]. Results from individual studies can be found in supplementary table 5.

[Insert Figures 2A, 2B, 2C and 2D about here].

2. Clinical supervision compared to within-group historical control (pre/post implementation)

Six studies evaluated the effect of clinical supervision on organisational outcomes by comparing post-implementation with pre-implementation [22,23,25,26,30,32]. While individual studies found clinical supervision had a positive effect on organisational outcomes, there were variable results across studies for burnout (figure 3A, 3B, 3C, 3D), job satisfaction, well-being, and workplace environment. Results from individual studies can be found in supplementary table 6.

[Insert Figures 3A, 3B, 3C and 3D about here].

3. Association between effective clinical supervision and organisational outcomes

There was preliminary evidence to suggest that effectiveness of clinical supervision may be negatively associated with burnout and positively associated with job retention (table 2). The association between effective clinical supervision and job satisfaction was unclear.

[Insert Table 2 about here].

Synthesis of five studies [37,39,41,42,52], including 1,046 participants, indicated that effectiveness of clinical supervision may be negatively associated with emotional exhaustion

and depersonalisation, but not associated with personal accomplishment. Three studies found a small to moderate association with emotional exhaustion [39,42,52] and four studies found small association with depersonalisation [37,39,41,42].

Synthesis of two studies [40,52], including 152 participants, indicated that effectiveness of clinical supervision may be positively associated with job retention. Both studies found a moderate association with job retention.

Synthesis of three studies [41-43], including 836 participants, indicated that the association between effectiveness of clinical supervision and job satisfaction was unclear. Two studies [41,42] found a small positive association and one study [43] found a small negative association. Results from individual studies are available in supplementary table 7.

4. Association between an effective supervisor and organisational outcomes

There was preliminary evidence to suggest that an effective supervisor may be negatively associated with burnout, and positively associated with job satisfaction (table 3).

[Insert Table 3 about here].

Synthesis of three studies, [39,41,48] including 901 participants, indicated that an effective supervisor may be negatively associated with depersonalisation but not associated with personal accomplishment. Two studies found a small association with depersonalisation [39,48]. The association between an effective supervisor and emotional exhaustion was

unclear, with two studies finding a small negative association [39,48] and one study finding a small positive association [41].

Synthesis of five studies [32,41,43,45,46], including 1128 participants, indicated that an effective supervisor may be positively associated with job satisfaction. Studies found a small to large association with job satisfaction. Results from individual studies are available in supplementary table 8.

Healthcare professionals' experiences of clinical supervision as it relates to organizational processes and outcomes (Qualitative findings)

Five studies, including two qualitative [49,50] and three mixed methods studies [51-53], explored the experiences of healthcare professional supervisees on clinical supervision as it relates to organisational outcomes. A total of 16 findings and their illustrations were extracted. Of the 16 findings, 14 were unequivocal and two were credible. The 16 findings were organised into four categories which were further deduced to two synthesised findings. Table 4 shows a summary of the qualitative findings.

[Insert Table 4 about here].

Synthesised Finding 1: Adequate clinical supervision mitigates the risk of burnout and facilitates staff retention, while inadequate clinical supervision can lead to stress and burnout

Health professionals indicated that if clinical supervision was adequate or if they felt supported the risk of experiencing burnout or leaving the workplace was less likely. Conversely, health

professionals, who felt that their supervision was inadequate, reported that clinical supervision had no positive impact or can lead to stress and burnout if they felt unsupported. This synthesised finding was developed from two categories comprising of seven unequivocal findings.

- *Category 1.1 Adequate clinical supervision mitigates the risk of burnout, while inadequate clinical supervision can lead to stress and burnout.*

Adequate supervision meant that health professionals experienced the opportunity to debrief challenging events with their supervisor and gain a better understanding of patient interactions which can be stressful, and cause burnout for some staff. However, participants who felt unsupported identified stress and burnout as the negative consequences. This category was supported by four findings:

1. Some respondents felt that inadequate supervision had no impact; however, others identified personal consequences in terms of stress and burnout, feeling unsupported and there being an impact on their work, the ward, and clients. (Unequivocal)
2. Supervision assisted them to manage the workplace stress and hence, reduce their risk of burnout. (Unequivocal)
3. Supervision was helpful for the worker to gain a greater understanding of the dynamics operating in the client interaction to ensure there were no negative impacts for the worker or the client. (Unequivocal)

4. Opportunity to debrief challenging events provided supervisees with validation of their feelings and consideration of different management strategies to reduce their distress. (Unequivocal)

- *Category 1.2 Implementation of effective clinical supervision facilitates staff retention and reduces their intention to leave.*

Participants reported that clinical supervision was a reflection that the health organisation valued their staff. Participants also indicated that supervisors encouraged staff to pursue career developments. These experiences enhanced job satisfaction and reduced staffs' intention to leave the healthcare organisation. This category was supported by three findings:

1. The implementation of clinical supervision as evidence that the health service management 'cared about' her and her colleagues and valued and wished to retain their workers. (Unequivocal)
2. Supervisees' responses illustrated that supervision did enhance job satisfaction and reduce workers' intention to leave. (Unequivocal)
3. The supervisor played an active role in encouraging staff to undertake career developing activities. (Unequivocal)

Synthesised Finding 2: Clinical supervision improves the work environment through boosting of staff morale, motivation to work, staff well-being and team relationships

Health professionals indicated that clinical supervision was valuable, led to increased motivation and enthusiasm at work, and provided not only reassurance to staff but also a safe

space for improved working relationships. This synthesised finding was developed from two categories comprising of seven unequivocal findings and two credible findings.

- *Category 2.1 Clinical supervision enhances team relationships through improved communication.*

Participants felt that clinical supervision offered an opportunity to enhance their ability to engage in difficult conversations with their team which is key in effective working relationships. This category was supported by two findings

1. Midwives felt the structure of a safe space for regular reflection offered them continual opportunities for self-development especially in terms of enhanced communication and improved working relationships. (Unequivocal)
2. Midwives used words such as ‘courage’, ‘confidence’ and ‘strength’ to describe how their clinical supervision sessions had fostered in them an improved ability to engage in difficult conversations at work. (Unequivocal)

- *Category 2.2 Clinical supervision promotes staff morale, motivation to work and well-being.*

Participants reported that having a clinical supervisor to support them and provide valuable feedback made them believe that they had a place within their organisation, increased their morale and enthusiasm at work, and improved their overall perception of their work environment. This category was supported by six findings:

1. Midwives described feeling more positive about the work environment with an increased desire to ‘give back’ to the unit. (Credible)

2. Prominent valuable outcomes of clinical supervision at the level of organization were the strengthened relationships with work colleagues, which on occasion was reported as a challenge for senior staff, and increased staff morale. (Credible)
3. Enthusiasm, growth and organisational commitment were identified by supervisors and supervisees. (Unequivocal)
4. Supervision kept workers motivated, interested, and engaged in their roles of delivering healthcare services. These features of supervision increased allied health workers' sense of connection to the employing organisation and decreased their intention to leave. (Unequivocal)
5. Receiving positive feedback was particularly valuable for workers (at the time of data collection) as they were experiencing high uncertainty in many areas including changes to their roles and the focus of the service. Feedback from supervisors provided reassurance, as well as a sense of stability amidst the evolving occupational landscape. (Unequivocal)
6. Supervision increased health professionals' sense of connection to the employing organisation, enabling supervisees to feel that they individually had a place within the organisation and therefore a sense of belonging to something greater than their immediate and often atomized local environment. (Unequivocal).

Integration of quantitative and qualitative evidence

Quantitative and qualitative findings in this review have been largely complementary and supportive of each other, especially on the impact of clinical supervision on burnout, staff well-being, job satisfaction, job retention and workplace environment.

Burnout:

Quantitative findings have provided preliminary evidence that effective clinical supervision and effective supervisor may be negatively associated with burnout. This was also supported by qualitative findings that showed that adequate clinical supervision mitigated the risk of burnout, and that inadequate clinical supervision lead to stress and burnout.

Staff well-being:

Quantitative findings from a single randomised controlled trial showed a large effect on reducing burnout and enhancing well-being. Qualitative studies supported this, showing that effective clinical supervision improved staff well-being.

Job satisfaction:

Although quantitative evidence from three studies showed that the association between effective clinical supervision and job satisfaction was unclear, evidence from four studies showed a positive association of an effective supervisor with job satisfaction. Qualitative findings supported this showing that effective clinical supervision strengthened team relationships and sense of belonging to the organisation, thereby enhancing job satisfaction. This was particularly true when the supervisor was effective, provided valuable feedback and encouraged staff to pursue career developments.

Job retention:

Evidence from two quantitative studies showed a moderate positive association of the effectiveness of clinical supervision with job retention. Similarly, qualitative studies showed that adequate clinical supervision facilitated staff retention.

Workplace environment:

Synthesis of quantitative evidence from 11 studies investigating the effect of clinical supervision, and six studies investigating post-implementation of clinical supervision with pre-implementation, showed variable results in regard to its effect on workplace environment. However, qualitative evidence highlighted that effective feedback from supervisors were considered valuable and improved supervisee perceptions of the work environment and their sense of belonging to the organisation.

In summary, both the quantitative and qualitative evidence highlight that effective clinical supervision and effective clinical supervisors may be associated with positive organisational outcomes, whereas, ineffective or inadequate clinical supervision and ineffective supervisors may have a negative impact on the well-being of the supervisee.

Discussion

This systematic review of 32 studies is the first known synthesis of quantitative and qualitative evidence to further our knowledge on the impact from, and experiences of, clinical supervision on organisational outcomes. Quantitative findings indicate that clinical supervision can have variable effects on organisational outcomes. The effectiveness of both the clinical supervision and the supervisor appear to influence this effect; effective clinical supervision is associated

with lower burnout and greater staff retention, and an effective supervisor is associated with lower burnout and greater job satisfaction. This is supported by the qualitative findings which show that healthcare professionals believe adequate clinical supervision can mitigate the risk of burnout, facilitate staff retention, and improve the work environment, while inadequate clinical supervision can lead to stress and burnout. Overall, qualitative synthesis highlights that the effectiveness of clinical supervision and supervisors can significantly influence the effect of clinical supervision on organisational outcomes.

Effective clinical supervision and effective supervisors may be pre-cursors for the realisation of beneficial effects of clinical supervision by healthcare organisations. This is consistent with a model of clinical supervision proposed by Gonge and Buss [42], where participation in effective clinical supervision is a pre-requisite to beneficial clinical supervision. While clinical supervision has become increasingly mandated in many healthcare organisations, through standard policies and procedures, the subsequent challenge lies in its effective and consistent implementation and uptake. This can be achieved in several ways. Organisations can adopt/utilise evidence-informed clinical supervision frameworks to guide supervision, such as the one recently developed by Rothwell and colleagues [54]. This review, based on evidence from 135 studies, encourages organisations to consider making supervision mandatory to increase the value placed on it, and provide protected time for supervisors and supervisees to engage with it. It also offers several practical strategies such as providing staff with both one-to-one and group supervision options, facilitating a person-centred supervision approach with clear boundaries, tasks, ground rules and record keeping processes, and provision of ongoing training to

supervisors and supervisees [54]. Implementation and uptake of clinical supervision can be completed by building a positive organisational culture that supports engagement in and uptake of clinical supervision [54], which could be regularly monitored through routine evaluations. Such evaluations will be critical to identify and respond to what clinical supervision strategies have worked, or not worked, for whom and why. Based on our work in this field, we believe that the organisational context can have an important role, and there is no one-size fits all approach when it comes to supporting the implementation and uptake of clinical supervision within organisations.

Healthcare organisations also need to support clinical supervisors to build and foster positive supervisory relationships with their supervisees. This has commonly been reported to be the single most important factor that influences the effectiveness of clinical supervision [11,54,55], and requires investment of both time and resources. Supervisors and supervisees can also be guided by evidence-informed principles that facilitate effective clinical supervision. For example, Martin and colleagues [11] provide several practical recommendations for supervisors and supervisees to enhance the effectiveness of clinical supervision, such as the development of a supervision contract, undertaking sessions at an optimal length and frequency, utilising different modes including telesupervision, evaluating supervision, and working on skills and abilities such as open communication, flexibility, trust and availability to foster a positive supervisory relationship [11]. This could take the form of professional development opportunities to upskill supervisors in evidence-informed supervision practices [55,56]. There is evidence from a longitudinal, multi-methods study to support the delivery of supervision

training in various modes such as videoconference, online and blended modes, thereby catering to those that can't access face-to-face training. In this study, participants knowledge and confidence in the provision of supervision increased after training, which was also sustained at three-months post-training across all the four modes. This success was attributed to the careful design and delivery of training across different modes [57].

This review found various methodological concerns across many studies reviewed, which is consistent with findings from a recent survey of 20 systematic reviews on clinical supervision reported between 1995 to 2019 [55]. Methodological concerns include predominance of ex post facto, cross-sectional, correlational designs, small sample sizes, over reliance on self-report measures, lack of psychometrically sound supervision measures, and lack of experimental and longitudinal designs [55]. Incomplete provision of information (on clinical supervision parameters) seems to continue to plague supervision research, as again found in the survey of supervision reviews [55], and in the systematic review reported here, making it hard to judge the full merit of the study or replicate it. There is a need for further rigorous high-quality studies in this area that use pluralistic research approaches where experimental investigation, randomisation, and data-driven case studies are used in conjunction with ex post facto, and cross-sectional designs [55]. Studies also need to better define the specifics of the clinical supervision *intervention* to allow replication and identification of the clinical supervision practices that are, or are not, effective for improving outcomes.

Limitations

The final review deviated from the protocol to also include group supervision, as many studies did not specify the type of supervision investigated. However, group supervision is commonly practiced in healthcare organisations and including these studies in this review likely improves the generalisability of our findings. Although the qualitative studies included were deemed to be of good quality, there were several shortcomings in the methodologies employed by the quantitative studies, especially the lack of randomised trials and absence of strategies to deal with confounding factors in cross-sectional studies. Although there were a variety of health professions represented in the studies included in this review, the majority of included studies were conducted in mental health disciplines such as psychology, counselling, and social work. This may limit the generalisability of the results to other disciplines and indicates the need for further research beyond mental health disciplines.

Conclusions

Clinical supervision can have a variable effect on healthcare organisational outcomes. The direction of this effect appears to be influenced by the effectiveness of both the clinical supervision provided and that of the clinical supervisor. This highlights the need for organisations to invest in high quality supervision practices if they wish to benefit from clinical supervision. Without such investment, there is a risk of policy-practice gaps in this area (i.e. while there may be policies to support clinical supervision in healthcare organisations, in practice it may not be implemented well). Ongoing further research, which grows the evidence

base for high quality clinical supervision and helps to unpack the *black box* of clinical supervision practices that have the most effect on organisational outcomes, is required.

Acknowledgements

The authors would like to thank Ms Esther Tian for assistance with the literature search.

References

1. Milne D. An empirical definition of clinical supervision. British Journal of Clinical Psychology. 2007; 46: 437-447. https://doi.org/10.1348/014466507X197415
2. Martin P, Kumar S & Lizarondo L. When I say clinical supervision. Med Edu. 2017; 51(9): 890-891.
3. Watkins CE. What do clinical supervision research reviews tell us? Surveying the last 25 years. Counselling Psychother Res. 2020; 188-206. https://doi.org/10.1002/capr.12287
4. Bambling M, King R, Raue P, Schweitzer R, Lambert W. Clinical supervision: Its influence on client-rated working alliance and client symptom reduction in the brief treatment of major depression. Psychother Res. 2006; 16(3):317-331.
5. Farnan JM, Petty LA, Georgitis E, Martin S, Chiu E, Prochaska M, et al. A systematic review: the effect of clinical supervision on patient and residency education outcomes. Acad Med. 2012; 87(4), 1 – 15.
6. Snowdon DA, Hau R, Leggat SG, Taylor NF. Does clinical supervision of health professionals improve patient safety? A systematic review and meta-analysis. Int J Qual Health Care. 2016; 28(4): 447-455.

7. Snowdon DA, Leggat SG, Taylor NF. Does clinical supervision of healthcare professionals improve effectiveness of care and patient experience? A systematic review. BMC Health Serv Res. 2017; 17:786.
8. Cutcliffe J, McFeely S. Practice nurses and their lived experience of clinical supervision. Brit J Nurs. 2001; 10(5):312-323.
9. Cummings GG, Tate K, Lee S, Wong CA, Paananen T, Micaroni SPM et al. Leadership styles and outcome patterns for the nursing workforce and work environment: A systematic review. Int J Nurs Studies. 2018; 85:19-60.
10. Hastings SE, Armitage GD, Mallinson S, Jackson K, Suter E. Exploring the relationship between governance mechanisms in healthcare and health workforce outcomes: a systematic review. BMC Health Serv Res. 2014; 14:479.
11. Martin P, Copley J, Tyack Z. Twelve tips for effective clinical supervision based on a narrative literature review and expert opinion. Med Teach. 2014; 36(3):201-207.
12. Hyrkäs K. Clinical supervision, burnout and job satisfaction among mental health nurses in Finland. Issues Mental Health Nurs. 2005;26(5):531-556.
13. Lizarondo L, Stern C, Carrier J, Godfrey C, Rieger K, Salmond S, Apostolo J, Kirkpatrick P, Loveday H. Chapter 8: Mixed methods systematic reviews. In: Aromataris E, Munn Z (Editors). JBI Manual Evid Synth. 2020. Available from https://synthesismanual.jbi.global. https://doi.org/10.46658/JBIMES-20-09. Accessed 10/12/2020.

14. Page MJ, McKenzie JE, Bossuyt PM, Boutron I, Hoffmann TC, Mulrow CD et al. The PRISMA 2020 statement: an updated guideline for reporting systematic reviews. BMJ. 2021; 372:n71 doi:10.1136/bmj.n71.
15. Martin P, Lizarondo L, Kumar S, Snowdon D. Impact of clinical supervision of health professionals on organizational outcomes: a mixed methods systematic review protocol. JBI Evid Synth. 2020;18(1):115-120. doi: 10.11124/JBISRIR-D-19-00017. PMID: 31464853.
16. EndNote 2015. Version 8.2. Thomson Reuters.
17. Joanna Briggs Institute Critical Appraisal tools. 2021. Available from https://jbi.global/critical-appraisal-tools. Accessed on 10/01/2021.
18. Wilson DB. Practical meta-analysis effect size calculator. (Online calculator). The Campbell Collaboration. 2015.
19. Cohen J. Statistical power analysis for the behavioral sciences (2nd ed.). 1988; Hillsdale, NJ: Erlbaum
20. Chen H, Cohen P, Chen S. How Big is a Big Odds Ratio? Interpreting the Magnitudes of Odds Ratios in Epidemiological Studies, Communications in Statistics—Simulation and Computation®. 2010; 39:4, 860-864, DOI: 10.1080/03610911003650383
21. Aromataris E, Munn Z (Editors). JBI Manual Evid Synth. JBI, 2020. Available from https://synthesismanual.jbi.global. https://doi.org/10.46658/JBIMES-20-01. Accessed on 10/02/2021.

22. Wallbank S. Effectiveness of individual clinical supervision of midwives and doctors in stress reduction: findings from a pilot study. Evidence-Based Midwifery. 2010;8(2):p. 65+.
23. Begat IBE, Severinsson EI, Berggren IB. Implementation of clinical supervision in a medical department: nurses' views of the effects. J Clin Nurs. 1997; 6:389-394.
24. Begat I, Ellefsen B, Severinsson E. Nurses' satisfaction with their work environment and the outcomes of clinical nursing supervision on nurses' experience of well-being – a Norwegian study. J Nurs Management. 2005; 13:221-230.
25. Berg A, Hansson UW, Hallberg IR. Nurses' creativity, tedium and burnout during 1 year of clinical supervision and implementation of individually planned nursing care: comparisons between a ward for severely demented patients and a similar control ward. J Adv Nurs. 1994; 20:742-749.
26. Berg A, Hallberg IR. Effects of systematic clinical supervision on psychiatric nurses' sense of coherence, creativity, work-related strain, job satisfaction and view of the effects from clinical supervision: a pre-post test design. J Psychiat Mental Health Nurs. 1999; 6:371-381.
27. Cooper-Nurse J. Hybrid Supervision: Impact on Burnout, Job Satisfaction, and Supervisory Relationship (Dissertation). Regent University. 2018.
28. Eklund M, Hallberg IR. Factors influencing job satisfaction among Swedish occupational therapists in psychiatric care. Scand J Caring Sciences. 2000; 14:162-171.

29. Fischer M, Mitsche M, Endler PC, Messenholl-Strehler E, Lonthaller H, Roth R. Burnout in physiotherapists: Use of clinical supervision and desire for emotional closeness or distance to clients. Int J Therapy Rehab. 2013; 20(11):550-558.

30. Hallberg IR. Systematic clinical supervision in a child psychiatric ward: Satisfaction with nursing care, tedium, burnout, and the nurses' own report on the effects of it. Archives Psychiat Nurs. 1994; 8(1):44-52.

31. Koivu A, Saarinen PI, Hyrkäs K. Who benefits from clinical supervision and how? The association between clinical supervision and the work-related well-being of female hospital nurses. J Clin Nurs. 2012; 21:2567-2578.

32. Livini D, Crowe TP, Gonsalvez CJ. Effects of supervision modality and intensity on alliance and outcomes for the supervisee. Rehab Psych. 2012;57(2):178-186.

33. McAuliffe E, Daly M, Kamwendo F, Masanja H, Sidat M, de Pinho H. The critical role of supervision in retaining staff in obstetric services: A three country study. PLOS One. 2013; 8(3):e58415.

34. Severinsson EI, Kamaker D. Clinical nursing supervision in the workplace – effects on moral stress and job satisfaction. J Nurs Management. 1999; 7:81-90.

35. Teasdale K, Brocklehurst N, Thom N. Clinical supervision and support for nurses: An evaluation study. J Adv Nurs. 2001;33(2):216-224.

36. Ben-Porat A, Itzhaky H. The contribution of training and supervision to perceived role competence, secondary traumatization, and burnout among domestic violence therapists. Clin Sup. 2011;30(1):95-108.

37. Berry S, Robertson N. Burnout within forensic psychiatric nursing: Its relationship with ward environment and effective clinical supervision? J Psychiat Mental Health Nurs. 2019; 26:212-222.

38. Best D, White E, Cameron J, Guthrie A, Hunter B, Hall K, Leicester S, Lubman DI. A model for predicting clinician satisfaction with clinical supervision. Alcoholism Treatment Quart. 2014;32(1):67-78.

39. Edwards D, Burnard P, Hannigan B, Cooper L, Adams J, Juggessur T, Fothergil A, Coyle D. Clinical supervision and burnout: the influence of clinical supervision for community mental health nurses. J Clin Nurs. 2006; 15:1007-1015.

40. Hussein R, Salamonson Y, Hu W, Everett B. Clinical supervision and ward orientation predict new graduate nurses' intention to work in critical care: Findings from a prospective observational study. Aust Critical Care. 2019; 32:397-402.

41. Hyrkas K. 2005. Clinical supervision, burnout, and job satisfaction among mental health and psychiatric nurses in Finland. Issues Ment Health Nurs, 26(5):531-56. 10.1080/01612840590931975.

42. Gonge H, Buus N. Model for investigating the benefits of clinical supervision in psychiatric nursing. A survey study. Int J Mental Health Nurs. 2011; 20:102-111.

43. Kavanagh DJ, Spence SH, Strong J, Wilson J, Sturk H, Crow N. Supervision practices in allied mental health: relationships of supervision characteristics to perceived impact and job satisfaction. Mental Health Serv Res. 2003;5(4):187-195.

44. Long CG, Harding S, Payne K, Collins L. Nursing and health-care assistant experience of supervision in a medium secure psychiatric service for women: implications for service development. J Psychiat Mental Health Nurs. 2014; 21:154-162.
45. Nathanson A. New social workers' satisfaction with supervision and with their job overall. Dissertation Abstracts International Section A: Humanities Social Sciences. 53(4-A):1276.
46. Schroffel A. How does clinical supervision affect job satisfaction? Clin Sup. 1999a;18(2):91-105
47. Severinsson EI, Hallberg IR. Systematic clinical supervision, working milieu and influence over duties: The psychiatric nurse's viewpoint – a pilot study. Int J Nurs Studies. 1996;33(4):394-406.
48. Webster L, Hackett RK. Burnout and leadership in community mental health systems. Admin Policy Mental Health. 1999;26(6):387-399.
49. White E, Butterworth T, Bishop V, Carson J, Jeacock J, Clements A. Clinical supervision: Insider reports of a private world. J Adv Nurs. 1998; 28(1); 185-192. 10.1046/j.1365-2648.1998.00743.x.
50. Ducat W, Martin P, Kumar S, Burge V, Abernathy L. Oceans apart, yet connected: Findings from a qualitative study on professional supervision in rural and remote allied health services. Aust J Rural health. 2016; 24(1): 29-35. http://dx.doi.org/10.1111/ajr.12192.

51. Love B, Sidebotham M, Fenwick J, Harvey S, Fairbrother G. Unscrambling what's in your head: A mixed method evaluation of clinical supervision for midwives. Women Birth. 2017; 30:271-281.

52. Saxby C. Clinical supervision, burnout and intent to leave: An Australian mixed methods study of community-based allied health professionals (Dissertation). The University of Queensland. 2016.

53. McCarron RH, Eade J, Delmage E. The experience of clinical supervision for nurses and healthcare assistants in a secure adolescent service: Affecting service improvement. J Psychiat Mental Health Nurs. 2018; 25(3):145-156. 10.1111/jpm.12447.

54. Rothwell C, Kehoe A, Farook S, Illing J. The characteristics of effective clinical and peer supervision in the workplace: a rapid evidence review. Final report. Newcastle University. Available from https://www.hcpc-uk.org/globalassets/resources/reports/research/effective-clinical-and-peer-supervision-report.pdf. Accessed on 6/5/2021.

55. Watkins CE. What do clinical supervision research reviews tell us? Surveying the last 25 years. Counsel Psychother Res. 2020; 188-206. https://doi.org/10.1002/capr.12287

56. Snowdon D, Kolic J, Taylor NF. Improvement in the short-term effectiveness of clinical supervsiion of physiotherapists who have taken part in a clinical supervision training program. Physiother Canada. 2020, e20190106.

57. Martin P, Kumar S, Abernathy L, Browne M. Good, bad or indifferent: a longitudinal multi-methods study comparing four modes of training for healthcare professionals in one Australian state. BMJ Open. 2018;8:e021264. doi: 10.1136/bmjopen-2017-021264.

Supporting Information Captions

- Appendix 1 – Search strategy
- Appendix 2 – Excluded studies
- Figure 1 – PRISMA
- Figure 2A, 2B, 2C & 2D – Studies where clinical supervision was compared to controls
- Figure 3A, 3B, 3C & 3D – Studies where clinical supervision was compared to within-group historical control (pre/post implementation)
- Table 1 – Study characteristics
- Table 2 - Synthesis of studies investigating association between effectiveness of clinical supervision and organisational outcomes
- Table 3 – Synthesis of results: association between an effective supervisor and organisational outcomes
- Table 4 – Summary of Qualitative Findings
- Supplementary Tables 1 to 4 – Critical appraisal checklists
- Supplementary Tables 5 to 8 – Quantitative results
- PRISMA checklist

 Click here to access/download;Figure;Figure 1_PRISMA.docx

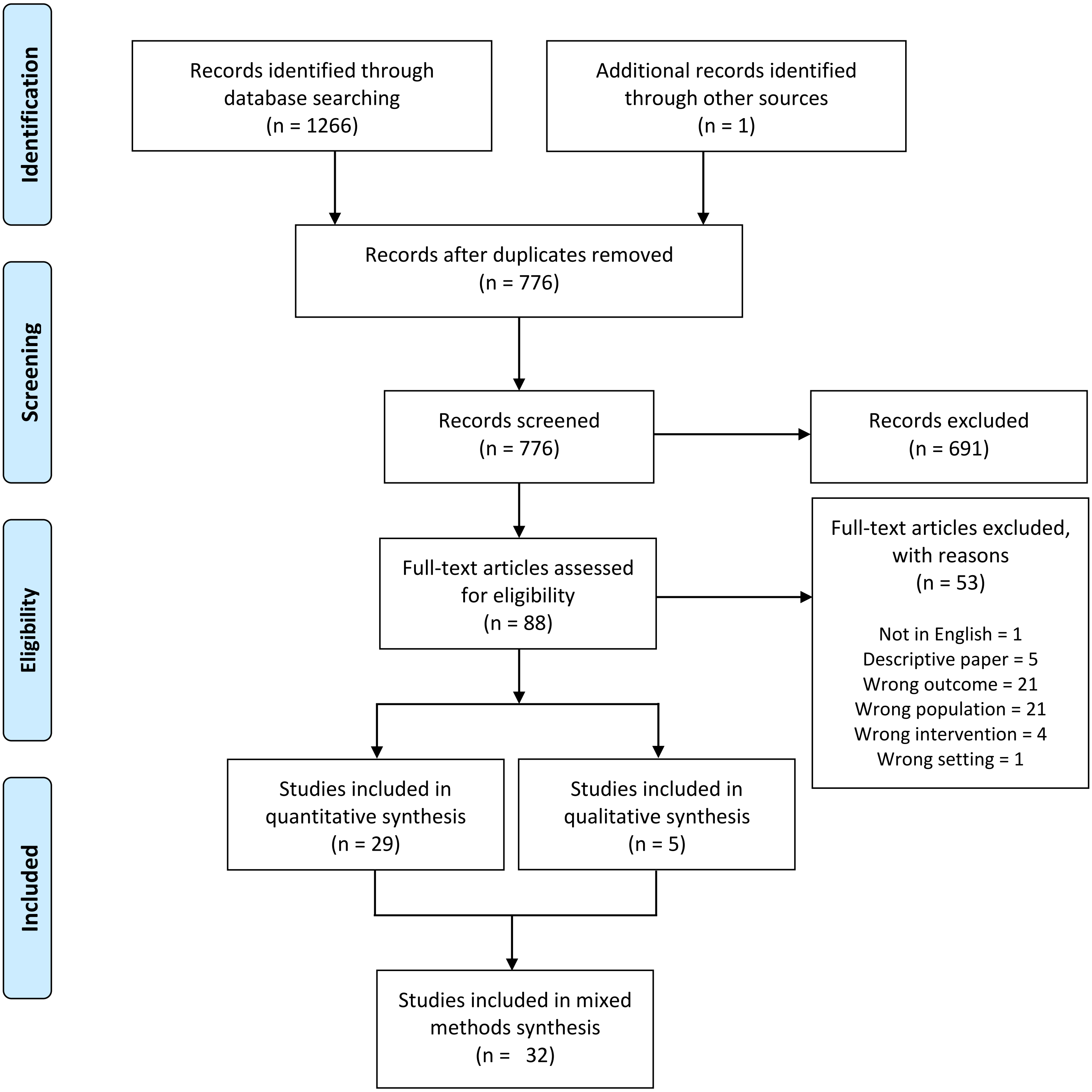

Figure 1: Flow diagram of included studies

From: Moher D, Liberati A, Tetzlaff J, Altman DG, The PRISMA Group (2009). *P*referred *R*eporting *I*tems for *S*ystematic Reviews and *M*eta-*A*nalyses: The PRISMA Statement. PLoS Med 6(7): e1000097. doi:10.1371/journal.pmed1000097

For more information, visit www.prisma-statement.org.

PRISMA checklist

Click here to access/download
Supporting Information
PRISMA Checklist.doc

Click here to access/download
Supporting Information - Compressed/ZIP File Archive
Supporting Information.zip
