# Supplementary material for: Impact of clinical supervision on healthcare organisational outcomes: A mixed methods systematic review
Source: PLoS One. 2021 Nov 19;16(11):e0260156. doi: 10.1371/journal.pone.0260156 (PMC8604366; doi:10.1371/journal.pone.0260156)
Supplement: Attachment — Submitted filename: Response_Letter_7Sep21.docx [file pone.0260156.s013.docx]

**Impact of clinical supervision on healthcare organisational outcomes: a mixed methods systematic review**

*PONE-D-21-20050*

***Response to reviewers’ comments***

We would like to thank the editors and reviewers for their valuable feedbacks on our manuscript. We have undertaken further revisions and have addressed all the issues raised by the reviewers. We feel that our manuscript has been strengthened by the reviewers’ insightful suggestions and additional comments.

Please find below a table of changes we have made to the manuscript following the review.

|  | **Reviewer 1 comments** | **Authors’ response** |
| --- | --- | --- |
| 1 | This comprehensive review explored an important topic. I enjoyed reading this manuscript which clearly synthesised the literature. I have some suggestions to enhance clarity. Thank you for synthesising and contributing to the evidence. | Thank you for this positive feedback. |
| 2 | Abstract:  L80 supervisor should be supervision? | No, this is indeed *supervisor* (not *supervision*) which is expanded upon in the body of the review. |
| 3 | Intro:  L93-94 needs reference L116-130 Rather than reporting each review, can you combine the outcomes and reference accordingly? | References now added.  Thank you for this suggestion. However, upon review, we were not able to combine the outcomes for these reviews as there are three categories of information provided:  1. Systematic review of leadership styles and outcomes patterns (lines 125-128)  2. Systematic review on the relationship between governance mechanisms in healthcare and health workforce outcomes (lines 128 -130)  3. Organisational outcomes in clinical supervision studies (lines 130 – 132).  As we have taken some applications from broader areas of research and applied it to the review in this context, we feel it is necessary to present these categories as they currently stand. |
| 4 | Methods: On the whole the methods were clearly described. **It would be beneficial to outline the philosophical framework (ie pragmatism?) for the meta-aggregative approach to help situate the reader at the outset.** This reference provides excellent context: Hannes, K. and Lockwood, C., 2011. Pragmatism as the philosophical foundation for the Joanna Briggs meta‐aggregative approach to qualitative evidence synthesis. Journal of advanced nursing, 67(7), pp.1632-1642.  L217 why were 3 levels of credibility assigned, and how was this decided? Quality appraisal was already conducted so it is not clear why there is an additional layer to this.    L241 Reference 21 does not relate to the meta-aggregative approach. This is perhaps why the above point requires clarifying  L241-255 How many authors conducted the qualitative synthesis? | This is indeed a wonderful suggestion and as such we acquiesce with the reviewer. Therefore, we have added information about the meta-aggregative approach as suggested (lines 249-252). We have now used the suggested reference.  Typically in systematic reviews, the methodological quality of the included literature is evaluated using critical appraisal processes. However, as this review was underpinned by JBI systematic review methodology, for meta-aggregative synthesis, in addition to critical appraisal, reviewers should assess the findings extracted from individual studies by assigning a level of credibility. The level of credibility is the degree of support each illustration (i.e. direct quotation of participant voice in included study) offers for the specific finding (theme or subtheme extracted from individual studies) it is associated with. This approach adds additional rigour to the systematic review methodology.  We have now changed the reference to Hannes & Lockwood 2011 as suggested.  The qualitative synthesis was undertaken by two reviewers. We have now included additional information reporting this process in lines 255-257. |
| 5 | Results: Quantitative findings were comprehensively reported.  L385 How was it determined that findings were credible or unequivocal? This was not described in the methods.  L406-409 Perhaps it is my lack of understanding regarding the “unequivocal” status, but finding 1 seems to indicate that supervision may or may not have an impact. So is the unequivocal finding that there is variability in supervisees’ perceptions of impact? | The JBI approach to data extraction for qualitative studies has set criteria for determining the level of credibility for each finding- this is described in lines 225-229. Additional information has also been included in lines 231-232 that data extraction, where assigning of levels of credibility also occurs, was performed by the reviewer with qualitative expertise and further validated by the primary reviewer. This approach adds additional rigour to the systematic review methodology.  Thank you for the opportunity to provide further clarity regarding this process. As per JBI methodology, levels of credibility apply to the findings (i.e. themes or subthemes) extracted from each included primary qualitative study, and not for the synthesised findings which emerged from the qualitative synthesis. This is described in lines 220-226. |
| 6 | Discussion: The discussion reads well and considers important aspects to translate the findings of this review.  L532-534 This sentence is not quite clear – it seems to state the obvious, as how can one experience beneficial supervision without participating in supervision?  L539 Which review?  L526-527 Is it inadequate supervision that leads to stress/burnout? Or the workload itself?  L604-605 Not sure that “The direction of this effect appears to be influenced by the effectiveness of both the clinical supervision provided and that of the clinical supervisor” when these data were not extracted from the study.  A number of interesting and practical points were made. It seems clear that clinical supervision is beneficial but concepts of cost to organisations (or individuals) was not considered. What are the barriers to implementing effective clinical supervision programs when there is clearly much evidence to support their benefits? | We acquiesce with the reviewer and this sentence has now been clarified (line 579-580).  This is the Rothwell et al. review, which has now been clarified (line 585).  This is a good point. Clinical supervision may provide the supervisee with a forum to work through issues such as workload. The findings presented here relate to inadequate clinical supervision (as presented in included papers), where there is missed opportunity for the supervisee to raise concerns about, and devise strategies to, help with issues such as increased workload.  Thank you for the opportunity to provide further clarity on this issue. We agree with the reviewer that data were not available to establish whether the effectiveness of clinical supervision/the clinical supervisor influenced the effect on organisational outcomes for studies that compared supervision with no supervision (or pre-implementation of supervision). However, findings from studies that investigated the association between the effectiveness of clinical supervision/the clinical supervisor and organisational outcomes found that these variables did influence organisational outcomes. These findings were also supported by findings from the qualitative studies which found that effectiveness of clinical supervision and the supervisor influenced organisational outcomes. Therefore, we believe that our conclusion summarizes the results of all studies included in this review.  We do, nevertheless, agree that use of the term ‘direction’ is misleading and have amended the sentence to remove this term (lines 652-653).  This is an important point and we have outlined in line 603 that supervisors and supervisees need to work on building a positive supervisory relationship, which requires time and resources (cost, infrastructure, staffing etc). The aim of our review was to determine the impact of clinical supervision on organisational outcomes. We have provided a case as well as strategies for organisations to support high quality clinical supervision of staff as it is likely to lead to better organisational outcomes. There is no one size fits all approach to implementing clinical supervision as it is complex, multi-factorial and depends on the context (see lines 595-598). We have described this in line 659 as the *black box* of clinical supervision as there are a lot of unknowns. |
|  | **Reviewer 2 comments** | **Authors’ response** |
| 1 | I have thoroughly enjoyed reading this systematic review. The authors are to be commended for their comprehensive review. I have only minor comments to align the manuscript to the PRISMA 2020 expanded checklist: PRISMA_2020_expanded_checklist.pdf (prisma-statement.org) | Thank you for this positive feedback. |
| 2 | Eligibility criteria - listed as inclusion criteria. **I would recommend changing the sub-title from inclusion criteria and using the term eligibility criteria** as per both 2009 and 2020 PRISMA Checklists. Comprehensive table of excluded studies and the respective reason is presented in Appendix 2. | We acquiesce with the reviewer and this sentence has now been clarified (line 155). |
| 3 | Areas for consideration: Search strategy – Comprehensive search strategy provided in Appendix 1. As this is a mixed methods review, it would be ideal to clarify if the search strategy was developed If the search strategy structure adopted a PICO-style approach/SPIDER/SPICER, then describe the final conceptual structure and any explorations that were undertaken to achieve it. | As indicated in the JBI guidance for undertaking a mixed methods systematic review, the PICO (for the quantitative component) and PICo (for the qualitative component) frameworks were used to define the inclusion criteria for this systematic review; this was reported in our published protocol. The development of our search strategy was informed by these frameworks. During testing of the strategy, it became apparent that using the terms corresponding to the Intervention/Phenomenon of Interest (i.e., clinical supervision) and outcomes of interest (i.e. organisational outcomes) yielded the most specific and sensitive search hits. Furthermore, while we are aware of SPIDER/SPICER, we also recognise there is no gold standard framework for use in systematic reviews. As outlined before, we chose PICO and PICo as per JBI systematic review methodology.  To further clarify this, we have added a sentence in the manuscript to indicate this approach to our search strategy (lines 174-176). |
| 4 | Study selection – reviewer roles during the screening have been specified. Please clarify additional PRISMA 2020 checklist (section 16) reporting recommendations: • Report any processes used to obtain or confirm relevant information from study investigators. • If abstracts or articles required translation into another language to determine their eligibility, report how these were translated | As per the reviewer’s suggestion, we have now specified that articles contained sufficient information to determine their eligibility and translation to another language was not required (lines 198-201). |
| 5 | Study risk of bias assessment - Quality assessment sub-header is stated, and reviewer involved noted. All studies were reviewed regardless of their methodological quality. **It is not clear why the Mixed Methods Assessment Tool (MMAT) was not utilised for example, for the included mixed methods studies.** The JBI Qualitative component is presented in supplementary Table 4. | As we followed the JBI approach to mixed methods systematic review, we adhered to JBI’s guidance on critical appraisal, i.e., the quantitative component of mixed methods primary studies were appraised using the JBI quantitative critical appraisal tool and the qualitative component appraised using the JBI qualitative tool. Furthermore, while we are aware of MMAT, we also recognise there is no gold standard critical appraisal tool for use in systematic reviews. As outlined before, we chose JBI critical appraisal tools, as these tools are published, widely used, and it follows JBI systematic review methodology. |
| 6 | Data extraction sub-heading is used instead of data collection process (PRISMA criteria 10) and Data items (PRISMA Criteria 11). **Consider revising the subheadings to align better with the PRISMA 2020 checklist.** The role and number of reviewers involved are reported within the manuscript. | This is a good suggestion, and we have changed the heading –data extraction—to data collection as suggested (line 213). |
| 7 | Certainty assessment is not overtly covered in the current manuscript (sections 15 and 22 of the PRISMA (2020) expanded checklist | Thank you for highlighting this point. There is currently no system to assess the certainty of evidence for outcomes obtained in a mixed methods systematic review. The GRADE approach is specific to quantitative outcomes and the ConQual/CerQual are specific to qualitative outcomes. Neither of these would be appropriate for mixed methods. Nevertheless, the process we have followed in this systematic review is a well-established and widely recognised process as indicated in the JBI manual/guidance for mixed methods systematic review. |
| 8 | Results: With respect to Supplementary Table 6 the Maslach burnout inventory scores are not individualised with respect to Livini (2012), is this an oversight or where they not separated in the research study. Perhaps worth clarifying. | This is a good point. The Maslach Burnout Inventory scores were not reported separately for each dimension. Instead, an overall score was provided for burnout. We have clarified this in a footnote in Supplementary Table 6. |
| 9 | There is an excellent summary of qualitative findings in Table 4**. To enhance the comparison of information in the main body of the manuscript and Table, it would be helpful to indicate the categorie**s: e.g., “Category 1.2 Implementation of effective clinical supervision facilitates staff retention and reduces their intention to leave…” stated on lines 418-419, but the table does not have numbered categories in column 2 of Table 4. | Synthesised findings and categories have been numbered in Table 4 so they are consistent with the reporting in the body of the manuscript. |
| 10 | The implications of the results for practice and policy are not overtly presented. Please make explicit recommendations for future research, as per section 23d of the PRISMA (2020) expanded checklist. | Thank you for the opportunity to provide further clarity on this. The positive impacts our review found were supported by limited number of studies, most with methodological flaws (see lines 620 onwards). Therefore, we were cautious of our interpretation and recommendations. Nevertheless, we have made a number of implications listed for policy, practice and future research:   - Organisations can adopt/utilise evidence-informed clinical supervision frameworks to guide supervision, such as one developed by Rothwell and colleagues (lines 583-585). - Strategies for organisations to implement this framework are provided in lines 586 to 591. - Information about the importance of a positive organisational culture and the value of evaluating these has been presented in lines 591 to 598. - Training recommendations have been provided for organisations in lines 610 to 618. - We have provided a number of recommendations for clinical supervisors and supervisors to strengthen their supervision in lines 605 to 610. - Future research recommendations have been provided in lines 628 to 633. |
|  | **Reviewer 3 comments** | **Authors’ response** |
| 1 | This manuscript presents a much-needed systematic review of the quantitative, qualitative, and mixed methods supervision literature capturing organizational outcomes in healthcare. The authors have conducted a methodologically sound, comprehensive review which will contribute to the interdisciplinary healthcare and supervision literature, and support practice. Additional information and clarity as noted below would be beneficial. | Thank you for this positive feedback. |
| 2 | Introduction In justifying the need for the study, the authors nicely present the gap in the supervision literature related to reviews of organizational outcomes, noting reviews typically focus on client or practitioner outcomes. **An additional comment/value statement related to why reviewing organizational supervision outcomes is important outside of it not being done would highlight the contribution of this manuscript** | This is a good suggestion, and we have now included additional information in line 136. |
| 3 | Methods: 1. given the definition provided, one would assume “group supervision” was led be a supervisor, and therefore “peer-supervision” only studies were not included. Can this be explicitly noted?  2. clarify “date of inception” pg. 7, line 176; 3. include date range of studies reviewed;  4. a brief definition of “pearling” on page 8 would be helpful. | This has now been explicitly noted in line 159. This is also in line with the clinical supervision definition that has been used in the introduction.  ‘Date of inception’ is an acceptable and common language used in systematic reviews. It means that the search for studies is performed from the date of when the database was established; in other words, we did not have any date restrictions.  We have now modified this sentence for clarity. (Lines 187-188) |
| 4 | Results: 5. I very much appreciated the detail presented, however, at times the narrative description is difficult to follow. A statement regarding the presentation (e.g., number of studies in specific categories are not mutually exclusive) would be helpful.  6. The sentence regarding setting is confusing as it seems to be reporting on both setting and profession. I found myself trying to understand if they were connected. Separate sentences per characteristic would make the meaning more clear, unless they are specifically connected in which case outlining the connection would be helpful.  7. In presenting the type of supervision, the total number of studies presented is 30, leaving me curious about the other two studies. Additionally, I was not sure why multi-professional supervision was separated out related to this characteristic?  8. For frequency and duration of supervision, can the number of studies included in the mean be reported explicitly? Or conversely the number that did not?  9. The number of participants represented in the studies as connected to methodological quality is helpful. **Including the number of participants included in all categories of impact would be useful (e.g., adding to control, and within-group sections on page 14 and 15).**  10. What definitions were used for “effective supervision” and “effective supervisor”?  11. For the synthesized finding, Category 2.1: was the finding about impact on teams specific to group supervision? Can this be noted? Midwives are also specifically noted in this finding, yet other disciplines are not noted at any other point in the Results. This leads to questions related to specifics of this discipline, and homogeneity of findings across the others. Can this please be clarified?  12. In the study protocol, authors identify the range of professions to be covered in this review. Aside from the above results related to midwives, and summary of nurses, psychology, social workers, and counsellors, there is little comment on the professions representation in this literature. Addressing this would be important. | As this systematic review followed best practice in the conduct and reporting of systematic reviews, when it came to presenting qualitative findings, we have presented narrative descriptions. Table 4 is available as a quick snapshot of the findings.  Thank you for this useful suggestion. We have now reported setting and profession characteristics separately, starting from line 294.  Thank you for the opportunity to clarify this issue. We have amended this section (lines 308 – 311) and it now includes the details of all 32 studies included in the review. We have removed reference to the study that reported multi-professional supervision (see line 310) and have amended Table 1 to reflect this change.  We present the range of frequency and duration of supervision across studies rather than a mean (in order to provide an overview of the data presented rather than a single measure of central tendency). In lines 314-315 we have stated that the number of studies that did not report on frequency or duration.  Thank you for this great suggestion. The number of studies and participants contributing to each category of impact has now been included in the sections comparing supervision with control and within group. See from page 15 onwards.  Quantitative studies included in this review investigated the association between the effectiveness of clinical supervision/the clinical supervisor and outcomes of interest. The effectiveness of clinical supervision/supervisor were measured in individual studies using different scales with different cut-offs to decide if supervision was effective or not. There are no agreed definitions for these terms in the literature. The literature focuses more on how clinical supervision can be made more effective than at that point in time (therefore, on a continuous scale, not binary).  Given the limited number of eligible studies and the heterogeneity of participants, there was no intention to analyse the findings by discipline but rather identify common ‘meanings’ or themes across studies. Analysing the findings of studies by disciplines will not allow meaningful synthesis to occur.  Thank you for identifying this issue. As we acquiesce with the reviewer, in lines 298-301, we have provided a more thorough description of the health professions covered in this review. We have also provided a description of the health professions represented in each synthesis (i.e. supervision vs. control, supervision within group comparison, association between effectiveness of clinical supervision and outcome, and association between effectiveness of supervisor and outcome).  We have refined our limitations section (line 645 onwards) to acknowledge the predominance of nursing and mental health disciplines in our review findings, and state that this may limit the generalisability of our results to other disciplines. |
|  | **Reviewer 4 comments** | **Authors’ response** |
| 1 | Thank you for the opportunity to review this interesting manuscript. Overall I found the work to be coherent and well-constructed. My major comment would be clarification in the title and throughout the manuscript that the work focuses on the post-professional clinical supervision literature, rather than the supervision literature as a whole (including pre-professional students). The methods section could also be reduced as much of this is described in the protocol paper but focus on the elements that were modified from the protocol. I have also made other suggestions throughout in the attached document. This will be a useful addition to the literature and thanks for the contribution. | Thank you for this positive feedback. |
| 2 | Tracked comments on pdf:  Line 3 – it may be helpful to include the focus of the paper in the title – postgraduate supervision.  Line 94 – It may be worth clarifying how supervision is beneficial to each of these groups and provide a reference or two to support it.  Line 95 – In some contexts it may not be quarantined and is balanced with patient care and administrative workloads. If this is to be retained it could be clarified by including reference to the post-graduate focus of the paper and potentially a reference or two to support it. This would help differentiate the rest of the Introduction from the wider supervision literature that includes pre-professional learners.  Line 94-98 – This sentence appears to narrow supervision to just the development of reflective practice skills. Maybe consider this work here:  Kilminster, S. M., & Jolly, B. C. (2000). Effective supervision in clinical practice settings: a literature review. Medical education, 34(10), 827-840.  Line 108 – Please clarify which are is being referred to here.  Line 113 – Again it may be helpful to clarify this in the context of post-graduate health professions practice.  Line 119 – Delete ‘the’ from ‘the healthcare’  Line 130 – May be useful to clarify this as the 'current' review or similar. It reads as though it is making reference to number 12.  Line 151 – Given the previously published protocol for the work, it may be better to summarise each section, with a focus on anything that was different to the published protocol. There is currently significant overlap between the protocol and the current work.  Line 280 – In this section it may be useful for the reader to understand when the studies were published.  Line 420 & 440 – As described above, I wonder if this may be better worded as 'Health professionals' or clarify that participants were those in the identified studies.  Line 440-442 – It appears that this finding is in the context of midwifery practice. It could be good to clarify in the lead in to the two findings so it is clear that it may not be a finding consistent across all health professions.  Line 491 – It would be good to include the measure of well-being here.  Line 518-520 – Again it would be good to clarify that this is referring to supervision of health professionals.  Line 533 – Could be useful to identify the context for this model, and also highlight that it is in the post-graduate space.  Line 550 – It would be great to include a few examples of elements that positively contribute to the organisational context. The reader may then be able to apply or modify these in their environment.  Line 564 – Is 'this' referring to the latter part of the previous sentence or the whole sentence?  Line 571 – It also suggests that the delivery mode may not be a significant consideration but may maximise the number of people who have access to the training. | This is a wonderful suggestion as it will help clarify the focus of this review. Based on the reviewer’s feedback, we have clarified this further throughout the review. We would still like to retain the existing title which is consistent with the published protocol, reflects the clinical supervision terminology in the published literature and as such aligns with the methodological processes which underpinned this review and is consistent with the evidence base.  Please see existing information on the benefits of clinical supervision from lines 109 to 114, supported by six references from the literature.  We acquiesce with the reviewer’s comments and as such have now clarified that the review pertains to post-qualification health professionals (line 95). Best practice in clinical supervision (as evidenced in the literature) advocates for quarantined time to undertake clinical supervision and health professionals are encouraged to prioritise and protect this time. Therefore the review team would like to retain this sentence as it stands.  Thank you for this suggestion. This has now been revised to broaden the scope to say, ‘supervisee’s skills and knowledge’ (line 97).  This has now been clarified (line 109).  The context has now been clarified (line 115).  Deleted (line 121).  This has now been clarified (line 132).  As this systematic review follows best practice standards in the conduct and reporting of systematic reviews (PRISMA 2020), there are critical elements that are required to be reported in the manuscript (a manuscript can be stand-alone document) and cannot be referred to elsewhere. Nevertheless, some overlap between the protocol and the full review is to be expected as they are both related to the same review. As most readers are likely to the read the full review independent of the protocol, the amount of detail provided here is expected to assist the readers in obtaining the complete picture.  This is a good suggestion and we have now included this information in lines 290 to 293.  As we have clarified further the context of this review in the introduction (ie. Pertaining to post-qualification health professionals), and throughout the manuscript, we expect this to address this concern.  We agree with the reviewer and this has now been clarified (line 484).  This is a good suggestion. However, given this section is a summary of the integration of quantitative and qualitative findings, the details of which have been elaborated in the previous section, we would like to retain this sentence as it is. Otherwise, it will be unnecessarily repetitive.  As per reviewer’s suggestion, this has now been clarified (line 564).  As per reviewer’s suggestion, this has now been clarified (line 578).  This is a great point and we have noted that organisations can create a positive culture and climate around supervision by adopting an evidence-informed framework (line 583), providing health professionals protected time for supervision (line 587), providing high quality training (line 610), plus several other practical recommendations from lines 583 to 618.  As per reviewer’s suggestion, this sentence has been re-phrased to improve clarity (lines 610 to 611).  As per reviewer’s suggestion, this has now been clarified (line 618). |

**Additional notes to the editor:**

We have made some non-tracked changes to the following tables (as these changes were not in response to reviewer comments but done to fix errors and to refine them):

- Table 2
- Table 3
- Supplementary Table 5

Table 2 and 3 were updated to include reference to the studies that were included in the reported synthesis. The letter 'a' has been removed from Supplementary Table 5 where we had reported 'Koivu 2012a' or 'Berg 1994a' - as this detail was only required during the drafting phase.

Kind regards,

Dr Priya Martin, Dr Lucylynn Lizarondo, A/Prof Saravana Kumar and Dr David Snowdon
